# Behavioral trainings and manipulations to reduce delay discounting: A systematic review

**DOI:** 10.3758/s13423-019-01629-2

**Published:** 2019-07-03

**Authors:** Hanneke Scholten, Anouk Scheres, Erik de Water, Uta Graf, Isabela Granic, Maartje Luijten

**Affiliations:** 1 Behavioural Science Institute, Radboud University, P.O. Box 9104, 6500 HE Nijmegen, The Netherlands; 2 Icahn School of Medicine at Mount Sinai, New York, NY USA

**Keywords:** Delay discounting, Temporal discounting, Trainings, Manipulations, Health behaviors, Trans-disease process

## Abstract

In everyday decision-making, individuals make trade-offs between short-term and long-term benefits or costs. Depending on many factors, individuals may choose to wait for larger delayed reward, yet in other situations they may prefer the smaller, immediate reward. In addition to within-subject variation in the short-term versus long-term reward trade-off, there are also interindividual differences in delay discounting (DD), which have been shown to be quite stable. The extent to which individuals discount the value of delayed rewards turns out to be associated with important health and disorder-related outcomes: the more discounting, the more unhealthy or problematic choices. This has led to the hypothesis that DD can be conceptualized as trans-disease process. The current systematic review presents an overview of behavioral trainings and manipulations that have been developed to reduce DD in human participants aged 12 years or older. Manipulation studies mostly contain one session and measure DD directly after the manipulation. Training studies add a multiple session training component that is not per se related to DD, in between two DD task measurements. Ninety-eight studies (151 experiments) were identified that tested behavioral trainings and manipulations to decrease DD. Overall, results indicated that DD can be decreased, showing that DD is profoundly context dependent and changeable. Most promising avenues to pursue in future research seem to be acceptance-based/mindfulness-based trainings, and even more so manipulations involving a future orientation. Limitations and recommendations are discussed to identify the mechanistic processes that allow for changes in discount rate and behavior accordingly.

## Glossary of acronyms in text and tables

ADHD: Attentional-deficit/hyperactivity disorder
ATM: Advisor-teller money manager
BMT: Brief motivational training
BS: Between-subject design
C: Control condition
CBT: Cognitive behavioral therapy
CDE: Cancer death experience
CLT: Construal level theory
CM: Contingency management
CO: Carbon monoxide
DD: Delay discounting
DSM: Diagnostic and Statistical Manual of Mental Disorders
E: Experimental condition
EFT: Episodic future thinking
EPT: Episodic past thinking
FDR: Fixed delayed reward
FF: Future focus
FIR: Fixed immediate reward
FITB: Fill-in-the-blank
HC: Healthy controls
HYP DD: Hypothetical delay discounting task
I: Investment
IGD: Internet gaming disorder
NI: No investment
NTF: Nontemporal focus
PF: Present focus
PR DD: Potentially real delay discounting task
PSA: Polysubstance dependent alcoholics
REAL DD: Real delay discounting task
SDT: Social delay discounting task
SFAS: Substance-free activity session
SFT: Semantic future thinking
STI: Sexually transmitted infection
VDR: Variable delayed reward
VIR: Variable immediate reward
WM: Working memory
WS: Within-subject design

Life is full of choices between options that are immediately rewarding and options that are only rewarding in the future. For example, shall I smoke a cigarette right now, or shall I refrain from smoking to stay healthier in the future? Individuals have the tendency to prefer immediate, smaller rewards over larger, but delayed rewards (Logue, 1988). This phenomenon is described as delay discounting (DD)—also referred to as temporal discounting or time discounting—and is often viewed as a measure of impulsive choice (e.g., Ainslie, 1975; Monterosso & Ainslie, 1999; Rachlin, 1989). DD refers to the decrease in the subjective value of a reward as the delay to its receipt increases (Ainslie, 1992; Critchfield & Kollins, 2001; L. Green & Myerson, 1993; Hamilton et al., 2015; Rachlin, 1989). The extent to which individuals discount the value of delayed rewards turns out to be associated with important health and disorder-related outcomes, and there is growing interest in trainings and manipulations that decrease heightened DD. The current systematic review presents an overview of behavioral trainings and manipulations that have been developed to reduce DD.

A DD task in which participants have to choose between a series of smaller–sooner (e.g., $5 today) and larger–later rewards (e.g., $10 in 2 weeks) is commonly used to assess these preferences (L. Green & Myerson, 2004; Stanger, Budney, & Bickel, 2013). The magnitude of the smaller–sooner reward and the delay preceding the larger reward are varied across choices in DD tasks, to determine an individual’s indifference point for each delay (Critchfield & Kollins, 2001). The indifference point, or subjective value, is defined as the magnitude of the smaller–sooner reward at which an individual shows no clear preference for either the smaller–sooner or later–larger reward. These indifference points/subjective values are used to define the rate at which individuals discount delayed rewards (Bickel, Jarmolowicz, Mueller, Koffarnus, & Gatchalian, 2012b; Scheres, de Water, & Mies, 2013a).

The choices in DD tasks can either be hypothetical, potentially real, or real. In hypothetical DD tasks, participants do not receive the rewards they choose, and they do not experience the waiting times (Scheres, de Water, et al., 2013a). In potentially real DD tasks, participants are informed that one choice will be selected at the end of the task, and participants are paid accordingly. This task relies on the assumption that participants will choose on each trial as if that trial is the one that will be selected. In real tasks, all chosen rewards are paid, and all delays are experienced during the test session (Scheres, de Water, et al., 2013a). Another method to assess DD, is the fill-in-the-blank (FITB) task (Chapman, 1996). In this task, participants answer only one question at each given delay, in comparison to the titrating procedures used in DD tasks (Weatherly & Derenne, 2011; Weatherly & Terrell, 2010). Specifically, for each choice participants indicate themselves what amount they are willing to accept immediately rather than having to wait for the full amount of the outcome that will be delayed for X amount of time (Weatherly & Derenne, 2011).

Frequently, unhealthy or problematic behaviors have a delayed effect on health—for example, smoking a cigarette right now has detrimental effects on one’s health in the long run. This has led researchers to believe that an individual’s tendency to make unhealthy or problematic choices is related to his or her discount rate. Supporting this belief, discount rate is highly correlated with a variety of health behaviors and disorders, with medium effect sizes across studies (Amlung, Petker, Jackson, Balodis, & MacKillop, 2016; Jackson & MacKillop, 2016; MacKillop et al., 2011). Specifically, increased DD rates are characteristic of maladaptive and unhealthy behaviors including alcohol dependence (e.g., Bobova, Finn, Rickert, & Lucas, 2009; Mitchell, Fields, D’Esposito, & Boettiger, 2005), drug dependence (e.g., Bickel, Landes, et al., 2011a; Kirby & Petry, 2004; Monterosso et al., 2007), gambling problems (e.g., Reynolds, 2006), tobacco use (e.g., Audrain-McGovern et al., 2009; Baker, Johnson, & Bickel, 2003; Bickel, Yi, Kowal, & Gatchalian, 2008; Fields, Leraas, Collins, & Reynolds, 2009), overeating (e.g., Amlung et al., 2016; Weller, Cook, Avsar, & Cox, 2008), attention-deficit/hyperactivity disorder (ADHD; Demurie, Roeyers, Baeyens, & Sonuga-Barke, 2012; Jackson & MacKillop, 2016; Patros et al., 2016; Scheres, Tontsch, Thoeny, & Kaczkurkin, 2010), conduct disorder (White et al., 2014), and risky sexual behaviors (Chesson et al., 2006). For example, as a group, individuals who smoke consistently show higher DD rates than controls do (Amlung & MacKillop, 2014; MacKillop et al., 2011). Thus, DD is consistently linked with a variety of problematic and unhealthy behaviors, and there is initial evidence from developmental studies that increased discounting rates contribute to the development and maintenance of these behaviors (Audrain-McGovern et al., 2009; Ayduk et al., 2000; Breaux, Griffith, & Harvey, 2016; Campbell & Von Stauffenberg, 2009; Khurana et al., 2013; Krishnan-Sarin et al., 2007; Passetti, Clark, Mehta, Joyce, & King, 2008; Sheffer et al., 2012; Stanger et al., 2012). Although more studies are needed to replicate these effects, Audrain-McGovern et al. (2009), for example, showed with a prospective longitudinal cohort study spanning midadolescence to young adulthood (ages 15–21 years old) that heightened baseline DD rates were a significant predictor of smoking initiation over time (11% increase in the odds of smoking uptake).

This body of evidence has led to the hypothesis that DD can be conceptualized as a trans-disease process that is shared across different disorders (Bickel et al., 2012b., ; Bickel, Quisenberry, Moody, & Wilson, 2015). Consequently, intervening in such a trans-disease process could be very promising for various reasons. First and foremost, if higher discount rates function as a behavioral marker of health behaviors and disorders, then manipulating discount rates might change multiple health behaviors and disorders as well (Koffarnus, Jarmolowicz, Mueller, & Bickel, 2013). Furthermore, it offers the opportunity to better understand and investigate comorbidity (i.e., the co-occurrence of two or more disorders). The presence of two or more disorders is not unexpected when both are originating from the same trans-disease process.

Interindividual differences in discounting rates are highly stable. This had led some to argue that the discounting rate should be viewed as a personality trait (Odum, 2011)—namely, someone’s relative DD rate is highly stable. At the same time, there is a growing number of studies that suggest that intraindividual differences in DD rate are substantial as well: Within individuals, DD rate changes as a function of contextual/situational factors (Bickel, 2015; Gray & MacKillop, 2015; Odum, 2011). Therefore, there is growing attention for trainings and manipulations that successfully target and decrease heightened DD (e.g., Bickel, Quisenberry, Moody, & Wilson, 2015; Koffarnus et al., 2013). Instead of focusing on specific disorders to identify trainings and manipulations that work, a more successful approach might be to develop trainings and manipulations that are effective across a variety of disorders.

While attention to the topic of reducing DD is growing, little is understood about effective ways to alter heightened discount rates. Understanding which trainings and manipulations are worth pursuing in the future, and which of those seem less effective in reducing DD, can help us optimize the application of this body of work. Although the literature provides a small number of important narrative reviews discussing the promise of DD trainings and manipulations (e.g., Bickel et al., 2015; Gray & MacKillop, 2015; Koffarnus et al., 2013; Lempert & Phelps, 2016), a systematic review was not performed at the time of our search. However, recently, a systematic review and meta-analysis was published on the same topic (Rung and Madden, b). Compared with the current systematic review, Rung and Madden (b) took a more methodological and meta-analytic approach to determine the efficacy of different methods to reduce DD, whereas we took a more theoretical approach to identify promising routes for future research. Furthermore, Rung and Madden (2018b) included a review and analysis of animal studies, studies with child populations, and studies before 1990, but did not report on possible effects of DD trainings or manipulations on behavior. The purpose of the current systematic review is to present an overview of behavioral trainings and manipulations available in the literature to reduce DD, and improve behavior accordingly, in human adolescent and adult participants.

Studies included in the review will be separated in two main categories. On the one hand, there are studies using manipulations: These studies manipulate the DD task (e.g., change wording in task) or add a priming procedure shortly before the DD task. Most of these studies include only one session and one moment of measuring DD rates. On the other hand, there are studies applying trainings: These studies add a training component that is not directly related to DD in between two DD task measurements. In most studies, these trainings are delivered in multiple sessions, but some studies include only one session of training. Within these two main categories, studies will be further classified based on the content of the training or manipulation. DD is the main outcome that is evaluated in the current systematic review, although secondary behavioral outcomes, such as smoking or eating behaviors, will also be discussed. The central research questions in this systematic review are whether there are effective ways of decreasing DD and whether there are associated effects on behavioral outcomes. This systematic review is a first step in systematically summarizing the research regarding decreasing DD. As this literature is rather diverse and large, we are not aiming to offer a coherent theoretical framework or compare effectiveness of studies by computing effect sizes. Yet we hope to identify promising routes for future research and classify overarching mechanistic processes that allow for changes in discount rate and behavior accordingly.

## Method

### Inclusion criteria

Studies were included in the systematic review if (1) they included human participants; (2) the participants were 12 years or older; (3) (one of) the outcome measure represented monetary DD; (4) a training or manipulation was employed; (5) the training or manipulation was behavioral in nature (no medication or neuromodulation studies are included); (6) the study aimed to decrease DD (instead of increase); (7) DD choices were made for the self instead of for others; (8) they included a training or manipulation that had training and/or clinical potential—for example, the mere manipulation of placing an individual in a certain environment (gambling vs. nongambling), or the experimental manipulation of reward magnitude or sign (gains vs. losses) were manipulation categories excluded from this review; (9) the sample size was >10; (10) they were published between 1990 and April 2017; and (11) were published in an English-language peer-reviewed journal. To reduce heterogeneity, we narrowed our search by including only studies using monetary outcomes that aimed to decrease DD via behavioral trainings or manipulations.

### Search strategies

Literature search and selection were carried out according to the Preferred Reporting Items for Systematic Reviews and Meta-Analyses (PRISMA; Moher, Liberati, Tetzlaff, Altman, & The PRISMA Group, 2009). A three-step procedure was used to identify relevant studies. First, we searched relevant databases (i.e., Pubmed, PsychInfo, and Web of Science) to identify studies that met inclusion criteria summarized above. All synonyms of the word delay discounting (i.e., delay discount*, temporal discount*, inter temporal choice and time discount*) and all possible variants of words like change and decrease (alter*, reduc*, manipulate*, train*, modify*, adjust*, transform*, convert*, reform*, diminish*, attenuate*, declin*, adapt*, improv*, amend*, ameliorat*, learn*, develop*) were entered in the databases. We included title, abstract, keywords, and topic as search areas. Second, to further identify relevant studies, the reference lists of all studies classified in step one were reviewed. Finally, we checked the reference lists of already existing reviews (i.e., Gray & MacKillop, 2015; Koffarnus et al., 2013; Lempert & Phelps, 2016) for additional studies.

### Search results

**Fig. 1:**
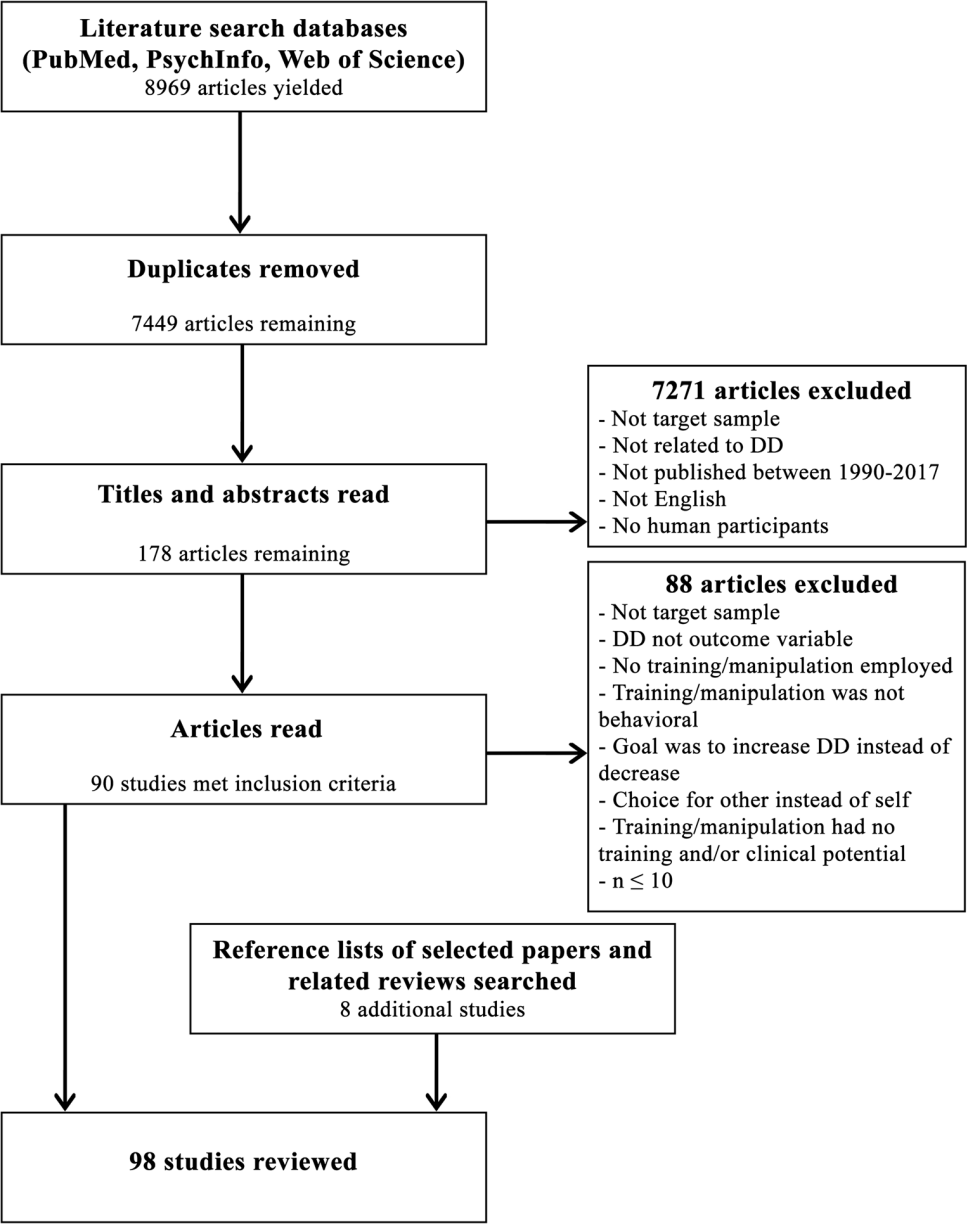
PRISMA flow diagram of literature search and selection process for inclusion in the systematic review

The literature search in Pubmed, PsychInfo, and Web of Science resulted in 8,969 hits (1,101; 6,584; and 1,284 hits, respectively). We detected 1,520 duplicates, and removing these resulted in 7,449 unique references. The reference lists of the identified studies yielded eight additional studies. No extra studies were identified when checking the reference lists of the already existing reviews. Authors H.S. and U..G independently determined whether the inclusion criteria were met by reading the titles and abstracts. We identified a total of 178 potentially relevant studies at this point. Next, all 178 full-text studies were screened using the same inclusion criteria, and this resulted in a final number of 98 studies relevant for the review. The selected studies cover a wide variety of behavioral trainings and manipulations that change DD in adolescents and adults. The whole literature search and selection process is displayed in Fig. 1.

## Results

### Characteristics of included studies

Characteristics and results of the 98 studies included in this systematic review are summarized in Table 1 and 2. Table 1 covers all studies applying a training, with a total of 19 studies (each covering one experiment; thus 19 experiments) published between 2008 and 2017. The majority (*n* = 14; 74%) of these studies included a clinical population, specifically smokers, individuals with Internet gaming disorder (diagnosed based on *Diagnostic and Statistical Manual of Mental Disorders, Fifth Edition* [DSM5] “condition for further study” symptoms), individuals dependent on marijuana, opioids, alcohol, cocaine, stimulants, or individuals on polysubstance and methadone maintenance. The remaining 26% (*n* = 5) of studies tested their training in healthy controls. Seven out of the 19 studies (37%) also reported effects of their training on actual behavior such as smoking or cocaine abstinence, in addition to results on DD.

**Table 1:** Characteristics and results of studies including trainings

Study	Experiment	Participants			Task			Result on DD	Secondary outcome
		Type	*N* (male)	Age	Type	Rewards	Delays		
**Contingency management (CM; main component)**									
Kurti & Dallery (2014)		Smokers	20 (13)	41.4	HYP DD	VIR, FDR	1w–10y	WS No interaction effect condition and time C: No exercise ➔ DD – E1: Exercise ➔ DD – Interaction effect condition and time? C: No CM ➔ DD ? E2: CM ➔ DD ?	Interaction effect condition and time E: Exercise + CM ➔ smoking ↓ E: No exercise + CM ➔ smoking ↓ E: Exercise + no CM ➔ smoking − C: No exercise + no CM ➔ smoking −
Landes et al. (2012)		Opioid-dependent patients	159 (83)	33.8	HYP DD	VIR, VDR	1d–25y	BS No interaction effect CM and time C: Buprenorphine ➔ DD ↓ E: Buprenorphine + CM ➔ DD ↓	–
Peters et al. (2013)		Marijuana-dependent individuals	93 (80)	26.1	REAL DD	VIR, FDR	7s–28s	BS Interaction effect condition and time C: CBT only ➔ DD ↑ E1: CM_abstinence_ only ➔ DD − E2: CBT + CM_adherence_ ➔ DD − E3: CM_abstinence_ + CBT ➔ DD −	–
Weidberg et al. (2015)		Treatment-seeking smokers	116 (44)	45.48	HYP DD	VIR, FDR	1d–25y	BS No interaction effect condition and time C: CBT only ➔ DD − E: CM + CBT ➔ DD − Interaction effect condition, time, and sex In women: C: CBT only ➔ DD − E: CM + CBT ➔ DD ↓ In men: C: CBT only ➔ DD − E: CM + CBT ➔ DD –	End of treatment: Interaction effect condition and time C: CBT only ➔ smoking abstinence – E: CM + CBT ➔ smoking abstinence ↑ Follow-up: No interaction effect condition and time C: CBT only ➔ smoking abstinence − E: CM + CBT ➔ smoking abstinence −
Yi et al. (2008)		Smokers	56 (36)	25.9	1. HYP DD (money) 1. HYP DD (cigarettes)	VIR, FDR	1w–25y	BS Interaction effect condition and time Money: C: Smoke as normal ➔ DD − E: CM ➔ DD ↓ Cigarettes: C: Smoke as normal ➔ DD − E: CM ➔ DD ↓	Interaction effect condition and time E: CM ➔ CO levels ↓ **No control condition**
Yoon et al. (2009)		Smokers	28 (19)	28.6	1. HYP DD (only money) 2. HYP DD (money and cigarettes)	VIR, FDR	1d–25y	BS Money: No interaction effect condition and time C: Control condition ➔ DD − E: CM ➔ DD − Money and cigarettes: Interaction effect condition and time C: Control condition ➔ DD − E: CM ➔ DD ↓	Interaction effect condition and time C: Control condition ➔ CO levels – E: CM ➔ CO levels ↓
**Money-management-based training**									
Black & Rosen (2011)		Cocaine and/or alcohol users	90 (45)	Adults	PR DD	VIR, VDR	7d–186d	BS Interaction effect condition and time C: Control condition ➔ DD ↑ E: Money management-based training ➔ DD −	Interaction effect condition and time C: Control condition ➔ cocaine abstinence ↓ E: Money management-based training ➔ cocaine abstinence −
DeHart et al. (2016)		HC	317 (120)	22.21	HYP DD	VIR, FDR	1w-25y	BS Interaction effect condition and time C: Abnormal psychology condition ➔ DD – E: Financial education condition ➔ DD ↓	–
**Brief motivational training in combination with substance-free activity session**									
Dennhardt et al. (2015)		Heavy drinking college students	97 (40)	20.10	HYP DD	–	–	BS No interaction effect condition and time C: Brief motivational training (BMT) + education session ➔ DD − E: BMT + Substance-free activity session (SFAS) ➔ DD −	–
Murphy et al. (2012)		Heavy drinking college students	82 (41)	18.5	HYP DD	VIR, VDR	7d–186d	BS No interaction effect condition and time C: BMT + relaxation training ➔ DD − E: BMT + SFAS ➔ DD −	Interaction effect condition and time C: BMT + relaxation training ➔ alcohol problems − E: BMT + SFAS ➔ alcohol problems ↓
**Cognitive behavioral therapy (CBT; main component)**									
De Wilde et al. (2013)		polysubstance dependent alcoholics (PSA)	37 (29)	31.61	HYP DD	VIR, VDR	2d–720d	WS No interaction effect condition and time E: CBT ➔ DD – **No control condition**	–
Secades-Villa et al. (2014)		Treatment-seeking smokers	80 (27)	38.90	HYP DD	VIR, FDR	1d–25y	WS No interaction effect condition and time E: CBT ➔ DD − **No control condition** End of treatment: No interaction effect smoking status and time E: Smoker ➔ DD − E: Abstainer ➔ DD − 12 month follow-up: Interaction effect smoking status and time E: Smoker ➔ DD − E: Abstainer ➔ DD ↓	–
**Acceptance-Based/Mindfulness-Based Trainings**									
Hendrickson & Rasmussen (2013)	Study2	HC	102 (29)	25.46	1. HYP DD (money) 2. HYP DD (food)	VIR, VDR	Money: 1d–365d Food: 1h–-20h	BS Money: No interaction effect condition and time C: Control condition ➔ DD − E: Mindfulness-based eating training ➔ DD − Food: Interaction effect condition and time C: Control condition ➔ DD – E: Mindfulness-based eating training ➔ DD ↓	–
Hendrickson & Rasmussen (2017)		HC	348 (134)	18.29	1. HYP DD (money) 2. HYP DD (food)	VIR, VDR	Money: 7d–186d Food: 0.5h–24h	BS Money: No interaction effect condition and time C1: Control condition ➔ DD − C2: Nutritional DVD ➔ DD − E: Mindfulness-based eating training ➔ DD − Food: Interaction effect condition and time C1: Control condition ➔ DD − C2: Nutritional DVD ➔ DD − E: Mindfulness-based eating training ➔ DD ↓	–
Morrison et al. (2014)		HC	30 (15)	21.5 (18–41)	HYP DD	VIR, FDR	1w–25y	BS Interaction effect condition and time C: Waitlist ➔ DD − E: Acceptance-based training ➔ DD ↓	–
Yao et al. (2017)		Individuals with Internet gaming disorder (IGD) / HC	25 (?) / 21 (?)	22.28 / 22.00	HYP DD	VIR, VDR	7d–186d	WS Main effect group Healthy control group ➔ DD − IGD group ➔ DD ↑ Interaction effect group and time In healthy control group: C: No training ➔ DD − In IGD group: E: Group behavioral training combining reality therapy and mindfulness meditation ➔ DD ↓	Interaction effect group and time In healthy control group: C: No training ➔ Internet addiction symptoms ↑ In IGD group: E: Group behavioral training combining reality therapy and mindfulness meditation ➔ Internet addiction symptoms ↓
**Working memory training**									
Bickel et al. (2011b)		Stimulant-dependent individuals in substance-abuse treatment	27 (20)	38.6	1. HYP DD 2. PR DD	VIR, FDR	HYP DD: 1d–25y PR DD: 1d–6m	BS Interaction effect condition and time C: Control condition ➔ DD − E: Working memory training ➔ DD ↓	–
Rass et al. (2015)		Methadone maintenance patients	56 (26)	43.4	1. HYP DD 2. REAL DD	VIR, FDR	HYP DD: 1d–25y REAL DD: 5s–80s	BS Hypothetical DD: No main or interaction effects C: Control condition ➔ DD − E: Working memory training ➔ DD − Real DD: Main effect time (real rewards) C: Control condition ➔ DD ↓ E: Working memory training ➔ DD ↓	–
**Visualization Training**									
Parthasarathi et al. (2017)		HC	48 (15)	24.6	PR DD	VIR, VDR	1d–180d	BS No interaction effect condition and time C: Control condition ➔ DD − E: Visualization training ➔ DD − Main effect condition at post-test C: Control condition ➔ DD − E: Visualization training ➔ DD ↑	–

*Note.* HYP DD = hypothetical delay discounting task; PR DD = potentially real delay discounting task; REAL DD = real delay discounting task; FITB = fill-in-the-blank task

VIR = variable immediate reward; FIR = fixed immediate reward; VDR = variable delayed reward; FDR = fixed delayed reward

WS = within-subject design; BS = between-subject design; E = experimental condition; C = control condition; HC = healthy controls

↓ = delay discounting decreased; ↑ = delay discounting increased; – = no changes in delay discounting

**Table 2:** Characteristics and results of studies including manipulations

Study	Experiment	Participants			Task			Result on DD	Secondary outcome
		Type	*N* (male)	Age	Type	Rewards	Delays		
**Future**									
**Episodic future thinking (EFT)**									
Benoit et al. (2011)		HC	12 (4)	27.3 (20.6–36.3)	HYP DD	FIR, VDR	30–360d	WS Main effect condition C: Control condition ➔ DD – E: EFT condition ➔ DD ↓ Interaction effect condition and emotional intensity Low emotional intensity: C: Control condition ➔ DD – E: EFT condition ➔ DD – High emotional intensity: C: Control condition ➔ DD – E: EFT condition ➔ DD ↓	–
Bulley and Gullo (2017)		HC	48 (15)	20.67	HYP DD	VIR, FDR	2d–365d	WS Main effect condition C: Control condition ➔ DD – E: EFT condition ➔ DD ↓	–
Cheng et al. (2012)	Exp. 1	HC	64 (34)	21.1	HYP DD	VIR, VDR	1w	BS Main effect condition C: Control condition ➔ DD – E: EFT condition ➔ DD ↓	–
Chiou & Wu (2016)		Smokers	90 (69)	31.4	HYP DD	FIR, VDR	1y	BS Main effect condition C1: Control condition ➔ DD – C2: Semantic future thinking (SFT) condition ➔ DD – E: EFT condition➔ DD ↓	Main effect condition on smoking during survey/smoking next week C1: Control condition ➔ smoking during survey/smoking next week – C2: SFT condition ➔ smoking during survey /smoking next week – E: EFT condition ➔ smoking during survey/smoking next week ↓ Mediation effect condition and DD on smoking during survey/smoking next week E: EFT ➔ DD ↓ ➔ smoking during survey/smoking next week ↓
Daniel et al. (2013b)		Obese individuals	26 (0)	26.43	HYP DD	VIR, FDR	1d – 2y	BS Main effect condition C: Control condition ➔ DD – E: EFT condition➔ DD ↓	Main effect condition on caloric intake C: Control condition ➔ caloric intake – E: EFT condition➔ caloric intake ↓
Daniel et al. (2013a)		Obese individuals	48 (0)	18 – 40	HYP DD	VIR, FDR	1d – 2y	WS Main effect condition C: Control condition ➔ DD – E: EFT condition➔ DD ↓ No interaction effect condition and group Lean individual group: C: Control condition ➔ DD – E: EFT condition ➔ DD ↓ Obese individual group: C: Control condition ➔ DD – E: EFT condition ➔ DD ↓	-
Daniel et al. (2016)		HC	81 (31)	26.07	HYP DD	VIR, FDR	1d–6m	BS Main effect condition C1: Control condition ➔ DD – C2: Episodic past thinking (EPT) condition ➔ DD – E: EFT condition➔ DD ↓	–
Dassen et al. (2016)		HC	95 (0)	20.45	HYP DD	VIR, VDR	7d–186d	BS Main effect condition C1: Food-related control condition ➔ DD – C2: General control condition ➔ DD – E1: Food-related EFT condition ➔ DD ↓ E2: General EFT condition ➔ DD ↓	Interaction effect condition and task content on caloric intake Food-related task: C1: Food-related control condition ➔ caloric intake – E1: Food-related EFT condition ➔ caloric intake ↓ General task: C2: General control condition ➔ caloric intake – E2: General EFT condition ➔ caloric intake –
Hu et al. (2017)		HC	22 (8)	24 (19-28)	HYP DD	FIR, VDR	1w–1y	WS Main effect condition C: Control condition ➔ DD – E: EFT condition➔ DD ↓	–
Kwan et al. (2015)		Amnestic patients / HC	6 (6) / 20 (12)	55.17 / 69.25	HYP DD	VIR, VDR	1w–10y	WS Main effect condition C: Control condition ➔ DD – E: EFT condition ➔ DD ↓	–
Lin & Epstein (2014)		HC	87 (45)	40.78	HYP DD	VIR, VDR	10d–70d	BS Main effect condition C: Control condition ➔ DD – E: EFT condition ➔ DD ↓	–
L. Liu et al. (2013)	Exp. 1	HC	32 (15)	20.62 (18–25)	PR DD	VIR, VDR	1w–1m	WS Main effect condition C: Control condition ➔ DD – E: Positive EFT condition ➔ DD ↓	–
	Exp. 2	HC	31 (16)	20.74 (18–25)	PR DD	VIR, VDR	1w–1m	WS Main effect condition C: Control condition ➔ DD – E: Negative EFT condition ➔ DD ↑	–
	Exp. 3	HC	30 (14)	21.48 (18–25)	PR DD	VIR, VDR	1w–1m	WS No main effect condition C: Control condition ➔ DD – E: Neutral EFT condition ➔ DD –	–
Palombo et al. (2015)		Amnestic patients / HC	9 (6) / 13 (6)	61.22 (45–85) / 65	HYP DD	FIR, VDR	2m–2y	WS Main effect condition C: Control condition ➔ DD – E: EFT condition ➔ DD ↓ Interaction effect condition and group In healthy control group: C: Control condition ➔ DD – E: EFT condition ➔ DD ↓ In amnestic patient group: C: Control condition ➔ DD – E: EFT condition ➔ DD –	–
Palombo et al. (2016)	Exp. 1	Amnestic patients / HC	9 (6) / 12(7)	58.78 (47-73) / 60.2	HYP DD	FIR, VDR	2m–2y	WS No main effect condition C: Control condition ➔ DD – E: EFT condition ➔ DD – Interaction effect condition and group In healthy control group: C: Control condition ➔ DD – E: EFT condition ➔ DD ↓ In amnestic patient group: C: Control condition ➔ DD – E: EFT condition ➔ DD ↑	–
	Exp. 2	Amnestic patients / HC	8 (5) / 12(6)	60.63 / 58.8	HYP DD	FIR, VDR	2m–2y	WS Main effect condition C: Control condition ➔ DD – E: EFT condition ➔ DD ↓ No interaction effect condition and group In healthy control group: C: Control condition ➔ DD – E: EFT condition ➔ DD ↓ In amnestic patient group: C: Control condition ➔ DD – E: EFT condition ➔ DD ↓	–
Peters & Büchel (2010)	Exp. 2	HC	30 (15)	25.4	PR DD	FIR, VDR	1d–233d	WS Main effect condition C: Control condition ➔ DD – E: EFT condition ➔ DD ↓	–
Sasse et al. (2015)		HC	23 (12)	24.96 (21–30)	PR DD	FIR, VDR	1d–190d	WS Main effect condition C1: Control condition ➔ DD – E1: Familiar EFT condition ➔ DD ↓ E2: Unfamiliar EFT condition ➔ DD ↓	–
Sasse et al. (2017)		HC	22 (9)	66.55	PR DD	FIR, VDR	1d–190d	WS No main effect condition C1: Control condition ➔ DD – E1: Familiar EFT condition ➔ DD – E2: Unfamiliar EFT condition ➔ DD –	–
Snider et al. (2016)		Alcohol-dependent individuals	50 (38)	41.15	HYP DD	VIR, FDR	1d–1y	BS Main effect condition C: Control condition ➔ DD – E: EFT condition ➔ DD ↓	Main effect condition on hypothetical alcohol purchase C: Control condition ➔ hypothetical alcohol purchase – E: EFT condition ➔ hypothetical alcohol purchase ↓
Stein et al. (2016)		Smokers	42 (24)	39.26	HYP DD	VIR, FDR	1d–1y	BS Main effect condition C: Control condition ➔ DD – E: EFT condition ➔ DD ↓	Main effect condition on cigarette self-administration C: Control condition ➔ cigarette self-administration – E: EFT condition ➔ cigarette self-administration ↓
Wu et al. (2017)	Exp. 1	HC	90 (48)	20.9	PR DD	FIR, VDR	1y	BS Main effect condition C1: Control condition ➔ DD – C2: SFT condition ➔ DD – E: EFT condition ➔ DD ↓	–
	Exp. 2	HC	90 (45)	20.2	PR DD	FIR, VDR	1y	BS Main effect condition C1: Control episodic thinking (ET) ➔ DD – C2: ET present-self ➔ DD – E: EFT condition ➔ DD ↓	–
**Connectivity to future (Self)**									
Bartels & Urminsky (2011)	Exp. 1	HC	141 (–)	Graduating seniors	PR DD	FIR, VDR	1w–1y	BS Main effect condition C: Low connectedness ➔ DD – E: High connectedness ➔ DD ↓	–
	Exp. 2	HC	118 (–)	18–29	PR DD	VIR, FDR	1w–1y	BS Main effect condition C: Low connectedness ➔ DD – E: High connectedness ➔ DD ↓	–
	Exp. 3	HC	97 (–)	Undergraduates	HYP FITB	–	Now–1y	BS Main effect condition C: Low connectedness ➔ DD – E: High connectedness ➔ DD ↓	–
	Exp. 4	HC	71 (–)	Adults	HYP FITB	–	1m/1y	BS Main effect condition C: Low connectedness ➔ DD – E: High connectedness ➔ DD ↓	–
Hershfield et al. (2011)	Exp. 2	HC	21 (6)	20.08	PR DD	VIR, VDR	10d–75d	BS No main effect condition C: Exposure to other ➔ DD – E: Exposure to future self ➔ DD –	–
Israel et al. (2014)		HC	436 (235)	25.27	HYP FITB	–	1w–1y	BS Picture priming: Main effect condition C: Basic prime ➔ DD ↓ E1: Vacation prime ➔ DD – E2: Older people prime ➔ DD ↓↓ Text priming: No main effect condition C: Basic prime ➔ DD – E1: Older people prime ➔ DD – E2: Vacation prime ➔ DD –	–
Joshi & Fast (2013)	Exp. 1	HC	73 (30)	33.11 (18–63)	HYP DD	FIR, VDR	1y	BS Main effect condition C: Low power ➔ DD – E: High power ➔ DD ↓	–
	Exp. 2	HC	59 (27)	19.95 (18–25)	PR DD	FIR, VDR	1y	BS Main effect condition C: Low power ➔ DD – E: High power ➔ DD ↓	–
Kuo et al. (2016)		HC	76 (28)	21.2	PR DD	FIR, VDR	1y	BS Main effect condition C: Present self condition ➔ DD – E: Future ideal self condition ➔ DD ↓	Main effect condition on ice cream intake C: Present self condition ➔ ice cream intake – E: Future ideal self condition➔ ice cream intake ↓ Mediation effect condition and DD on ice cream intake E: Future ideal self condition ➔ DD ↓ ➔ ice cream intake ↓ Main effect condition on amount of sugar in reward drink C: Present self condition ➔ amount of sugar – E: Future ideal self condition➔ amount of sugar ↓
Pronin et al. (2008)	Exp. 4	HC	140 (–)	College students	PR DD	FIR, FDR	2.5m–5m	BS Main effect condition C: Choosing for self ➔ DD – E1: Choosing for self in future ➔ DD ↓ E2: Choosing for another person ➔ DD ↓ E3: Choosing for self with reduced salience of emotions ➔ DD ↓	–
Sheffer et al. (2016)		HC	1,122 (583)	34.0	HYP DD	VIR, VDR	7d–186d	BS Main effect condition C1: Nontemporal focus condition ➔ DD – C2: Present focus condition ➔ DD – E: Future focus condition ➔ DD ↓	–
**Construal Level Manipulation**									
Kelley & Schmeichel (2015)		HC	118 (28)	21.19 (18–43)	HYP DD	FIR, VDR	3m	BS **ABSTRACTION** Main effect condition C: Control condition ➔ DD – E: Mortality salience condition ➔ DD ↓	–
Kim et al. (2012)	Exp. 4	HC	200 (90)	33.9	HYP FITB	–	3m	BS Main effect condition C: Large distance condition ➔ DD – E: Short distance condition ➔ DD ↓	–
	Exp. 5	HC	187 (86)	19.79	HYP FITB	–	1m	BS Main effect condition C: Large distance condition ➔ DD – E: Short distance condition ➔ DD ↓	–
Kim et al. (2013)	Exp. 1a	HC	70 (33)	25.47	HYP DD	VIR, FDR	1y	BS **CONCRETIZATION** Main effect condition C: High-level construal ➔ DD – E: Low-level construal ➔ DD ↓	−
	Exp. 1b	HC	81 (39)	23.60	HYP DD	VIR, FDR	1y	BS **CONCRETIZATION** Main effect condition C: High-level construal ➔ DD – E: Low-level construal ➔ DD ↓	−
	Exp. 2	HC	102 (35)	36.25	HYP DD	VIR, FDR	1y	BS **CONCRETIZATION** Main effect condition C: High-level construal ➔ DD – E: Low-level construal ➔ DD ↓	−
Li et al. (2016)		Internet addicts / HC	55 (39) / 55 (25)	19 / 19	HYP DD	VIR, FDR	6m	BS **ABSTRACTION** Main effect condition C: Low-level construal ➔ DD – E: High-level construal ➔ DD ↓ No interaction effect condition and group In healthy control group: C: Low-level construal ➔ DD – E: High-level construal ➔ DD ↓ In internet addicts group: C: Low-level construal ➔ DD – E: High-level construal ➔ DD ↓	−
Malkoc et al. (2010)	Exp. 1a	HC	102 (–)	College students	HYP FITB	–	3d-10d	BS **ABSTRACTION** Main effect condition C: Low-level construal ➔ DD – E: High-level construal ➔ DD ↓	−
	Exp. 1b	HC	522 (−)	College students	HYP FITB	−	3d−10d	BS **ABSTRACTION** Main effect condition C1: Control condition ➔ DD – C2: Low-level construal ➔ DD – E: High-level construal ➔ DD ↓	−
	Exp. 2	HC	117 (−)	College students	HYP FITB	−	4w−10w	BS **ABSTRACTION** Main effect condition C: Low-level construal ➔ DD – E: High-level construal ➔ DD ↓	−
	Exp. 3	HC	231 (−)	College students	HYP FITB	−	3m−1y	BS **ABSTRACTION** Main effect condition C: Low-level construal ➔ DD – E: High-level construal ➔ DD ↓	−
	Exp. 4	HC	171 (−)	College students	HYP FITB	−	3m−1y	BS **ABSTRACTION** Main effect condition C: Low-level construal ➔ DD – E: High-level construal ➔ DD ↓	−
**Social Factors**									
**Social Context**									
Bickel, Jarmolowicz, Mueller, Franck, et al. (2012a)		Smokers and hazardous-to-harmful drinkers	796 (358)	31.31	HYP DD	VIR, VDR	10d–75d	WS Main effect condition C: Me now, me later ➔ DD – E1: Me now, we later ➔ DD ↓ E2: We now, we later ➔ DD ↓	−
Charlton et al. (2013)	Exp. 1	HC	32 (–)	College students	HYP DD	VIR, VDR	7d–186d	WS Main effect condition C: Choosing for self ➔ DD – E: Choosing for group ➔ DD ↓	−
	Exp. 2	HC	32 (–)	College students	HYP DD	VIR, VDR	7d–186d	WS Main effect condition C: Choosing for self ➔ DD – E: Choosing for group ➔ DD ↓	−
	Exp. 3	HC	108 (–)	College students	HYP DD	VIR, VDR	7d−186d	WS Main effect condition C: Choosing for self ➔ DD – E: Choosing for group ➔ DD ↓	−
Yi et al. (2010)		HC	57 (26)	33.09	HYP DD	VIR, FDR	1d−25y / 1w−5y	WS No main effect condition C: Choosing for self ➔ DD – E: Choosing for group ➔ DD – Interaction effect condition and sex In males: C: Choosing for self ➔ DD – E: Choosing for group ➔ DD ↓ In females: C: Choosing for self ➔ DD ↓ E: Choosing for group ➔ DD –	−
**Social influence**									
Senecal et al. (2012)	Exp. 2	HC	80 (27)	22.2	PR DD	VIR, VDR	1d−180d	BS design/WS analyses Main effect condition C: Interest rate instruction ➔ DD – E: Financial guide ➔ DD ↓ **No direct comparison between 2 conditions** Interaction effect condition and time C: Interest rate instruction ➔ DD – E: Financial guide ➔ DD ↓ **No direct comparison between 2 conditions**	−
	Exp. 3	HC	20 (5)	26	PR DD	VIR, VDR	1d–180d	WS Main effect condition E: Financial guide ➔ DD ↓ **No control condition** Interaction effect condition and time E: Financial guide ➔ DD ↓ **No control condition**	–
	Exp. 4	HC	64 (24)	21	PR DD	VIR, VDR	1d–180d	BS design/WS analyses Main effect condition C: Impatient peer-generated advice ➔ DD – E: Patient peer-generated advice ➔ DD ↓ **No direct comparison between 2 conditions**	–
**Emotion**									
Augustine & Larsen (2011)	Exp. 1	HC	70 (21)	19.66	HYP DD	VIR, VDR	7–186d	BS No main effect condition C: Negative condition ➔ DD – E: Positive condition ➔ DD – Interaction effect condition and neuroticism Low neuroticism: C: Negative condition ➔ DD – E: Positive condition ➔ DD – High neuroticism: C: Negative condition ➔ DD ↓ E: Positive condition ➔ DD –	–
	Exp. 2	HC	67 (18)	19.34	HYP DD	VIR, VDR	7–186d	BS No main effect condition C: Negative condition ➔ DD – E: Positive condition ➔ DD – Interaction effect condition and neuroticism Low neuroticism: C: Negative condition ➔ DD ↓ E: Positive condition ➔ DD – High neuroticism: C: Negative condition ➔ DD – E: Positive condition ➔ DD –	–
Berndsen & van der Pligt (2001)	Exp. 1	HC	83 (−)	College students	HYP FITB	–	1y–4y	BS Main effect condition C: High optimism ➔ DD – E: Low optimism ➔ DD ↓	−
	Exp. 2	HC	81 (−)	College students	HYP FITB	–	1y–4y	BS Main effect condition C: High optimism ➔ DD – E: Low optimism ➔ DD ↓	−
Berry et al. (2014)		HC	185 (78)	20.88	HYP DD	VIR, FDR	1d–25y	BS Main effect condition C1: Built environments ➔ DD – C2: Geometric shapes ➔ DD – E: Natural environment ➔ DD ↓	−
Berry et al. (2015)		HC	43 (17)	22.53	HYP DD	VIR, FDR	1d−25y	BS Main effect condition C: Built environment ➔ DD – E: Natural environment ➔ DD ↓	−
Callan et al. (2014)	Exp. 1	HC	381 (164)	30.82	HYP DD	VIR, FDR	Lab sample + 1^st^ online sample: 1d–730d 2^nd^ online sample:7d–365d	BS Interaction condition and victim derogation Unjust (faith in justice threatened): C: Low victim derogation ➔ DD – E: High victim derogation ➔ DD ↓ Just (faith in justice): C: Low victim derogation ➔ DD – E: High victim derogation ➔ DD –	−
	Exp. 2	HC	238 (133)	31.10	HYP DD	VIR, FDR	7d–365d	BS Main effect condition C: No drug dealer condition ➔ DD – E: Drug dealer condition ➔ DD ↓	–
DeSteno et al. (2014)		HC	75 (32)	19 (18–23)	PR DD	VIR, VDR	1w–6m	BS Main effect condition C1: Neutral condition ➔ DD – C2: Happiness condition ➔ DD – E: Gratitude condition ➔ DD ↓	–
Dickens & DeSteno (2016)		HC	105 (26)	19.31	PR DD	VIR, VDR	1w–6m	WS Main effect condition E: Daily gratitude ➔ DD ↓ **No control condition**	−
Guan et al. (2015)		HC	27 (7)	19.3	HYP DD	VIR, VDR	1m	WS Main effect condition C: Negative condition ➔ DD – E1: Neutral condition ➔ DD ↓ E2: Positive condition ➔ DD ↓↓	−
Hirsh et al. (2010)		HC	137 (38)	20.1 (18–25)	HYP DD	VIR, VDR	1w–1y	BS No main effect condition C1: Control condition ➔ DD – C2: Positive affect induction ➔ DD – E: Negative affect induction ➔ DD – Interaction effect condition and extraversion Low extraversion: C1: Control condition ➔ DD – C2: Positive affect induction ➔ DD – E: Negative affect induction ➔ DD – Medium and high extraversion: C1: Control condition ➔ DD ↓ C2: Positive affect induction ➔ DD – E: Negative affect induction ➔ DD ↓	−
Huang et al. (2016)	Exp. 1	HC	80 (41)	20.77	PR DD	FIR, FDR	1m	BS Main effect condition C: Control condition ➔ DD – E: Nostalgia condition ➔ DD ↓	−
	Exp. 6	HC	186 (99)	38.53	PR DD	FIR, FDR	30d	BS Interaction effect condition and repeatability C1: Repeatable control condition ➔ DD – C2: Unrepeatable control condition ➔ DD – E1: Repeatable nostalgia condition ➔ DD – E2: Unrepeatable nostalgia condition ➔ DD ↓	−
Ifcher & Zarghamee (2011)		HC	69 (36)	Undergraduate students	PR FITB	–	1d-56d	BS Main effect condition C: Neutral condition ➔ DD – E: Positive condition ➔ DD ↓	−
W. Liu & Aaker (2007)	Exp. 4	HC	80 (38)	20.91	HYP FITB	–	Spend it/Short-term saving/ Long-term saving	BS Interaction effect condition and simulation No cancer death experience (CDE): C: No mental simulation of CDE ➔ DD – E: Mental simulation of CDE ➔ DD ↓ CDE: C: No mental simulation of CDE ➔ DD ↓ E: Mental simulation of CDE ➔ DD ↓	−
Luo et al. (2014)		HC	22 (13)	33.6	PR DD	VIR, VDR	1d–56d	WS Main effect condition C: Neutral condition ➔ DD – E1: Happy prime ➔ DD – E2: Fearful prime ➔ DD ↓	−
Pyone & Isen (2011)	Exp. 3	HC	95 (42)	College students	HYP DD	FIR, VDR	4w–6w	BS Interaction effect condition and reward amount $5 reward: C: Neutral condition ➔ DD – E: Positive condition ➔ DD – $15 or more reward: C: Neutral condition ➔ DD – E: Positive condition ➔ DD ↓	−
	Exp. 4	HC	117 (–)	College students	HYP DD	FIR, FDR	A few weeks	BS Interaction effect condition and reward amount $5 reward: C: Neutral condition ➔ DD – E: Positive condition ➔ DD – $15 reward: C: Neutral condition ➔ DD – E: Positive condition ➔ DD ↓	−
	Exp. 5	HC	44 (–)	College students	HYP FITB	–	1m	BS Main effect condition C: Neutral condition ➔ DD – E: Positive condition ➔ DD ↓	−
	Exp. 6	HC	50 (–)	College students	HYP FITB	–	3d–10d	BS Main effect condition C: Neutral condition ➔ DD – E: Positive condition ➔ DD ↓	−
Quisenberry et al. (2015)		HC	408 (228)	30 (25–37)	1. HYP DD 2. Sexual DD (SDT)	1. VIR, FDR 2. Without condom, with condom	1. 1d–5y 2. 1h–3m	BS 1. No effects 2. Interaction effect condition and scenario Least attractive scenario: C: Positive condition ➔ DD – E1: Negative no regret condition ➔ DD ↓ E2: Negative regret condition ➔ DD ↓ Most attractive scenario: C: Positive condition ➔ DD – E1: Negative no regret condition ➔ DD – E2: Negative regret condition ➔ DD ↓ Least sexually transmitted infection (STI) partner scenario: C: Positive condition ➔ DD – E1: Negative no regret condition ➔ DD – E2: Negative regret condition ➔ DD ↓ Most STI partner scenario: C: Positive condition ➔ DD – E1: Negative no regret condition ➔ DD ↓ E2: Negative regret condition ➔ DD ↓	−
Raeva et al. (2010)		HC	57 (30)	23.07	PR DD	VIR, VDR	1d–2m	WS Main effect condition C: Partial feedback ➔ DD – E1: Regret feedback ➔ DD – E2: Rejoicing feedback ➔ DD ↓	−
van der Wal et al. (2013)	Exp. 1	HC	47 (22)	20.23	PR DD	FIR, VDR	90d	BS Main effect condition C: Urban environment ➔ DD – E: Nature environment ➔ DD ↓	−
	Exp. 2	HC	67 (19)	20.03	PR DD	VIR, VDR	7d–91d	BS Main effect condition C1: Control condition ➔ DD – C2: Urban environment ➔ DD – E: Nature environment ➔ DD ↓	−
	Exp. 3	HC	43 (17)	31.84	PR DD	VIR, VDR	7d–236d	BS Main effect condition C: Urban environment ➔ DD – E: Nature environment ➔ DD ↓	−
**Framing**									
**Bundling**									
Białaszek and Ostaszewski (2012)		HC	54 (23)	21.31 (18–32)	HYP DD	VIR, VDR	1m–10y	WS Small rewards: No main effect condition C: Single small reward ➔ DD – E: Sequence of small rewards ➔ DD – Large rewards: Main effect condition C: Single large reward ➔ DD ↓ E: Sequence of large rewards ➔ DD –	−
Hofmeyr et al. (2011)		Smokers + nonsmokers	30 (16) + 30 (16)	20.97 + 21.23	REAL DD	VIR, FDR	1d–10d	BS Smokers: Main effect condition C: Free condition ➔ DD – E1: Suggested condition ➔ DD – E2: Forced condition ➔ DD ↓ Nonsmokers: No main effect condition C: Free condition ➔ DD – E1: Suggested condition ➔ DD – E2: Forced condition ➔ DD –	−
Kirby & Guastello (2001)	Exp. 1	HC	72 (27)	Undergraduates	PR DD	VIR, FDR	1d–46d	WS Main effect condition C: Free-linking condition ➔ DD – E1: Suggested-linking condition ➔ DD – E2: Imposed-linking condition ➔ DD ↓	−
	Exp. 2	HC	38 (12)	Undergraduates	PR DD	VIR, FDR	7d	WS Main effect condition C: Free-linking condition ➔ DD – E1: Suggested-linking condition ➔ DD ↓ E2: Imposed-linking condition ➔ DD ↓↓	−
**Time framing**									
Dai & Fishbach (2013)	Exp. 1	HC	98 (48)	College students	PR DD	FIR, FDR	3d–50d	BS Main effect condition C1: Near-future condition ➔ DD – C2: Distant-future condition ➔ DD – E: Waiting condition ➔ DD ↓	−
	Exp. 2a	HC	157 (73)	College students	PR DD	FIR, FDR	2d–40d	BS Main effect condition C1: Near-future condition ➔ DD – C2: Distant-future condition ➔ DD – E: Waiting condition ➔ DD ↓	−
	Exp. 2b	HC	145 (–)	College students	PR DD	FIR, FDR	5d–45d	BS Main effect condition C1: Near-future condition ➔ DD – C2: Distant-future condition ➔ DD – E: Waiting condition ➔ DD ↓	−
	Exp. 3	HC	239 (128)	College students	PR DD	FIR, FDR	6d–48d	BS Main effect condition C: Short-wait ➔ DD – E: Long-wait ➔ DD ↓	−
	Exp. 4	HC	234 (87)	College students	PR DD	FIR, FDR	6d–48d	BS Main effect condition C: Short-wait ➔ DD – E: Long-wait ➔ DD ↓	−
DeHart & Odum (2015)		HC	76 (31)	21	HYP DD	VIR, FDR	1w–25y	BS Main effect condition C1: Temporal distance condition ➔ DD ↓ E1: Temporal distance condition (days) ➔ DD – E2: Specific date condition ➔ DD ↓↓	−
Dshemuchadse et al. (2013)		HC	42 (14)	24.1	HYP DD	VIR, VDR	1–14d	WS Main effect condition C: Temporal distance condition ➔ DD – E: Specific date condition ➔ DD ↓	−
Ebert & Prelec (2007)	Exp. 3	HC	121 (59)	22.2	HYP DD	VIR, FDR	1d–1y	BS Interaction effect condition and future time Near future: C: No focus ➔ DD – E: Time focus ➔ DD ↓ Far future: C: No focus ➔ DD ↓ E: Time focus ➔ DD –	−
	Exp. 4	HC	218 (107)	21.1	HYP DD	VIR, FDR	1d–1f8m	BS Interaction effect condition and future time Near future: C: No focus ➔ DD – E: Time focus ➔ DD ↓ Far future: C: No focus ➔ DD ↓ E: Time focus ➔ DD –	−
Lempert et al. (2016)		HC	60 (26)	23.43	PR DD	VIR, VDR	7d–180d	WS No main effect condition C: Temporal distance condition ➔ DD – E: Specific date condition ➔ DD – Main effect emotional arousal for delayed reward Low subjective value delayed reward (low emotional arousal): C: Temporal distance condition ➔ DD – E: Specific date condition ➔ DD – High subjective value delayed reward (high emotional arousal): C: Temporal distance condition ➔ DD ↓ E: Specific date condition ➔ DD ↓	−
Klapproth (2012)		Substance abuse disorder / HC	30 (23) / 30 (9)	36.9 / 31.3	HYP DD	VIR, VDR	7–186 d	BS Main effect condition C: Temporal distance condition ➔ DD – E: Specific date condition ➔ DD ↓ Interaction effect condition and group In healthy control group: C: Temporal distance condition ➔ DD – E: Specific date condition ➔ DD – In substance use disorder group: C: Temporal distance condition ➔ DD – E: Specific date condition ➔ DD ↓	−
LeBoeuf (2006)	Exp. 1a	HC	356 (–)	College students	HYP FITB	–	3m–10m	BS Main effect condition C: Temporal distance condition ➔ DD – E: Specific date condition ➔ DD ↓	−
	Exp. 1b	HC	240 (–)	College students	HYP FITB	–	2m–18m	BS Main effect condition C: Temporal distance condition ➔ DD – E: Specific date condition ➔ DD ↓	−
	Exp. 2	HC	253 (–)	College students	HYP FITB	VIR, VDR	–	BS Main effect condition C: Temporal distance condition ➔ DD – E: Specific date condition ➔ DD ↓	−
	Exp. 3	HC	86 (–)	College students	HYP DD	VIR, VDR	2m–23m	BS Main effect condition C: Temporal distance condition ➔ DD – E: Specific date condition ➔ DD ↓	−
Rabinovich et al. (2010)	Exp. 2	HC	94 (30)	36 (20–70)	PR DD	VIR, FDR	1m–7m	BS No main effect condition C: Short-term time perspective ➔ DD – E: Long-term time perspective ➔ DD – Interaction effect condition and attitudes towards saving Negative attitude towards saving: C: Short-term time perspective ➔ DD – E: Long-term time perspective ➔ DD – Positive attitude towards saving: C: Short-term time perspective ➔ DD – E: Long-term time perspective ➔ DD ↓	−
Read et al. (2005)	Exp. 1	HC	90 (–)	College students	HYP DD	FIR, FDR	2m–36m	BS Main effect condition C1: Temporal distance condition (days) ➔ DD – C2: Temporal distance condition (months) ➔ DD – E: Specific date condition ➔ DD ↓	−
	Exp. 2	HC	160 (-)	College students	HYP FITB	–	2m–36m	BS Main effect condition C: Temporal distance condition (months) ➔ DD – E: Specific date condition ➔ DD ↓	−
	Exp. 3	HC	60 (–)	College students	PR DD	FIR, FDR	2m–36m	BS Main effect condition C: Temporal distance condition (months) ➔ DD – E: Specific date condition ➔ DD ↓	−
	Exp. 4	HC	90 (–)	College students	HYP DD	FIR, FDR	2m–60m	BS Main effect condition C: Temporal distance condition (months) ➔ DD – E1: Temporal distance (months) + specific date condition ➔ DD – E2: Specific date condition ➔ DD ↓	−
Zauberman et al. (2009)	Exp. 3	HC	190 (–)	College students	HYP FITB	–	1m–3m	BS No main effect condition C: Control condition ➔ DD – E: Duration-priming condition ➔ DD – Interaction effect condition and time horizon 1 month time horizon: C: Control condition ➔ DD – E: Duration priming condition ➔ DD ↓ 3 month time horizon: C: Control condition ➔ DD ↓ E: Duration priming condition ➔ DD ↓	−
**Reframing of rewards**									
Appelt et al. (2011)	Exp. 1	HC	607 (152)	37.51	HYP DD	VIR, VDR	3m	BS Main effect condition C: Delay condition ➔ DD – E: Acceleration condition ➔ DD ↓	−
	Exp. 2	HC	279 (84)	39.82	HYP DD	VIR, VDR	3m	BS Main effect condition C: Delay condition ➔ DD – E: Acceleration condition ➔ DD ↓	−
Bickel, Wilson, et al. (2016b)	Emotion manipulation as well	HC	599 (383)	28.61	HYP DD	FIR, FDR	–	BS Main effect condition C: Hidden-zero condition ➔ DD – E: Explicit-zero condition ➔ DD ↓ Main effect condition (emotion) C: Negative income narrative ➔ DD ↑ E1: Neutral income narrative ➔ DD – E2: Positive income narrative ➔ DD –	−
Fassbender et al. (2014)	Exp. 1	HC	42 (17)	22.4	PR DD	VIR, VDR	7d–56d	WS Main effect condition C: Rounded decimal condition ➔ DD – E: Nonzero decimal condition ➔ DD ↓	−
	Exp. 4	HC	183 (92)	35.5	HYP DD	VIR, VDR	7d–56d	BS Main effect condition C: Rounded decimal condition ➔ DD – E: Nonzero decimal condition➔ DD ↓	−
	Exp. 5	ADHD / HC	25 (12) / 40 (23)	18.6 / 17.6	PR DD	VIR, VDR	7d–56d	BS Main effect condition C: Rounded decimal condition ➔ DD – E: Nonzero decimal condition ➔ DD ↓ No interaction effect condition and group In healthy control group: C; Rounded decimal condition ➔ DD – E: Nonzero decimal condition ➔ DD ↓ In ADHD group: C: Rounded decimal condition ➔ DD – E: Nonzero decimal condition ➔ DD ↓	−
Grace & McLean (2005)		HC	24 (14)	24.9	HYP DD	VIR, FDR	1m–10y	WS Main effect condition C1: Baseline condition ➔ DD – E1: Delay condition ➔ DD ↓ E2: Accelerate condition ➔ DD ↓↓	−
Jiang et al. (2014)	Exp. 1a	HC	209 (55)	21.3	HYP DD	VIR, FDR	1w–4y	BS Main effect condition C: Pure gain condition ➔ DD – E: Upfront loss + gain condition ➔ DD ↓	−
	Exp. 1b	HC	106 (54)	22.5	HYP DD	VIR, FDR	1w–4y	WS Main effect condition C: Pure gain condition ➔ DD – E: Upfront loss + gain condition ➔ DD ↓	−
	Exp. 2a	HC	171 (82)	20.8	HYP DD	VIR, FDR	1w–4y	BS Main effect condition C: Pure gain condition ➔ DD – E: Upfront gain + gain condition ➔ DD ↓	−
	Exp. 2b	HC	103 (40)	21.5	HYP DD	VIR, FDR	7w–9y	BS Main effect condition C: Pure gain condition ➔ DD – E: Upfront loss + gain condition ➔ DD ↓	−
	Exp. 2c	HC	104 (61)	21.1	HYP DD	VIR, FDR	3w–4y	WS Main effect condition C: Pure gain condition ➔ DD – E: Upfront loss + gain condition ➔ DD ↓	−
Magen et al. (2008)	Exp. 1	HC	112 (14)	33.7	HYP DD	VIR, VDR	7d–140d	BS Main effect condition C: Hidden-zero condition ➔ DD – E: Explicit-zero condition ➔ DD ↓	−
	Exp. 2	HC	57 (13)	32.1	PR DD	VIR, VDR	7d–140d	BS Main effect condition C: Hidden-zero condition ➔ DD – E: Explicit-zero condition ➔ DD ↓	−
Magen et al. (2014)	Exp. 1	HC	182 (89)	35.66	PR DD	VIR, VDR	7d–140d	BS Main effect condition C: Hidden-zero condition ➔ DD – E: Explicit-zero condition ➔ DD ↓	−
	Exp. 2	HC	23 (10)	27.5	PR DD	VIR, VDR	7d–140d	WS Main effect condition C: Hidden-zero condition ➔ DD – E: Explicit-zero condition ➔ DD ↓	
Radu et al. (2011)	Exp. 2	HC	47 (19)	18.8 (17–22)	HYP DD	VIR, VDR	7d–140d	WS Main effect condition C: Hidden-zero condition ➔ DD – E: Explicit-zero condition ➔ DD ↓	−
Read et al. (2013)	Exp. 1	HC	373 (134)	36	HYP DD	VIR, VDR	1y–3y	BS Main effect condition C: Amount condition ➔ DD – E: Interest-rate condition ➔ DD ↓	−
	Exp. 2	HC	630 (252)	35	HYP DD	VIR, VDR	1y-10y	BS Main effect condition (analyses without interest-rate condition) C1: No investment (NI) Amount condition ➔ DD – C2: NI Interest-total condition ➔ DD – E1: Investment (I) Amount condition ➔ DD ↓ E2: I Interest-total condition ➔ DD ↓	−
	Exp. 3	HC	219 (99)	37	HYP DD	VIR, VDR	1y–10y	BS No main effect condition C: Amount condition ➔ DD – E1: Interest-rate condition ➔ DD – E2: Composite condition ➔ DD –	−
Weatherly et al. (2010)		HC	648 (207)	19.53	HYP FITB	–	1w–10y	BS Main effect condition C: Won money condition ➔ DD – E: Owed money condition ➔ DD ↓	−
Weatherly & Derenne (2011)		HC	156 (28)	21.20	1. HYP DD 2. HYP FITB	1. VIR, FDR 2. –	6m–10y	WS Main effect condition C: Won money condition ➔ DD – E: Owed money condition ➔ DD ↓	−
Weatherly & Terrell (2010)		HC	177 (78)	19.66	HYP FITB	–	1w–10y	BS Main effect condition C: Won money condition ➔ DD – E: Owed money condition ➔ DD ↓	−
Weber et al. (2007)	Exp. 1	HC	176 (74)	38	PR DD	VIR, VDR	3m	BS Main effect condition C: Delay condition ➔ DD – E: Acceleration condition ➔ DD ↓	−
	Exp. 2	HC	112 (?)	CDS Virtual-Lab volunteers	PR DD	VIR, VDR	3m	BS Main effect condition C: Delay condition ➔ DD – E: Acceleration condition ➔ DD ↓	−
	Exp. 3	HC	89 (?)	CDS Virtual-Lab volunteers	PR DD	VIR, VDR	3m	BS Main effect condition C: Delay condition ➔ DD – E: Acceleration condition ➔ DD ↓	−

*Note.* HYP DD = hypothetical delay discounting task; PR DD = potentially real delay discounting task; REAL DD = real delay discounting task; HYP FITB = hypothetical fill-in-the-blank task; PR FITB = potentially real fill-in-the-blank task; REAL FITB = real fill in the blank task

VIR = variable immediate reward; FIR = fixed immediate reward; VDR = variable delayed reward; FDR = fixed delayed reward

WS = within subject design; BS = between subject design; E = experimental condition; C = control condition; HC = healthy controls

↓ = delay discounting decreased; ↑ = delay discounting increased; – = no changes in delay discounting

All studies using manipulations to change DD are displayed in Table 2, with a total of 79 studies published between 2001 and 2017. A total of 132 experiments were conducted within these 79 studies. Most experiments (*n* = 118; 89%) included healthy controls as their target population; however, 14 experiments (11) included clinical populations (sometimes in combination with a healthy control sample). These clinical populations included smokers, individuals with Internet gaming disorder, obese individuals, alcohol-dependent or substance-dependent individuals, and ADHD or amnestic patients. Of the experiments, 5% (*n* = 6) reported effects of the manipulations on actual behavior, such as caloric intake, in addition to the effect on DD. For clarification purposes, we divided the manipulations in four main categories broadly covering the variety of manipulations in the literature and discuss them accordingly: future, social factors, emotion, and framing. Please note that this division is purely subjective, and some studies combine multiple manipulation categories.

There are far more manipulation experiments available in the literature (*n* = 132; 87%), compared to trainings (*n* = 19; 13%). Almost all experiments included adults; only two experiments (Fassbender et al., 2014, Study 5; Radu, Yi, Bickel, Gross, & McClure, 2011, Study 2) also included participants younger than 18. Inspecting the samples more carefully, it becomes clear that more than a third of all manipulation experiments (*n* = 52; 39%) has tested its manipulation in healthy control college student samples with a higher percentage of female than male participants (>60%). In the training experiments, the percentage of healthy control college student samples with more female than male participants was much lower (*n* = 4; 21%).

With regard to measuring DD as the main outcome variable, most experiments employed either a hypothetical DD task (*n* = 77; 51%), a potentially real DD task (*n* = 46; 30%) or a hypothetical FITB task (*n* = 22; 15%). A small number of experiments used a real DD task (*n* = 2; 1%), a potentially real FITB task (*n* = 1; 1%) or combined two methods within one experiment (*n* = 3; 2%). One experiment combined a real DD and a hypothetical DD task, one experiment combined a potentially real DD task with a hypothetical DD task, and another experiment combined a hypothetical DD task with a hypothetical FITB task.

### General results

Results of the current review show that 26% (*n* = 5) of the experiments evaluating the effects of trainings find the expected reductions in DD, whereas 58% (*n* = 11) found null results or unexpected increases in DD. The remainder three studies (16%) found mixed results; these studies found the expected reductions in DD on a substance-related DD task, but not on a monetary DD task. Of the experiments that included a secondary behavioral outcome (*n* = 7) all found the expected positive effects on behavior.

Regarding the studies evaluating the effects of manipulations on DD, 86% (*n* = 114) found the expected reductions in DD, 13% (*n* = 17) found null results or unexpected increases in DD and 1% (*n* = 1) found mixed results (i.e., only effects in healthy control group, not in amnestic patients). These results indicate that DD can be decreased, showing that DD is profoundly context dependent and changeable.

### Trainings

#### Contingency management

Contingency management (CM) is a well-researched and effective behavioral training to increase drug abstinence across substance-dependent disorders (Stanger et al., 2013). It promotes abstinence by delivering material incentives contingent on biochemically verified abstinence (Higgins, Silverman, & Heil, 2008). Simply put, participants are paid for not using drugs. CM attempts to directly influence decision-making processes by shifting preferences for immediate rewards to delayed rewards (Stanger et al., 2013). Six studies examined the effect of a CM training, four studies included smokers (Kurti & Dallery, 2014; Weidberg, Landes, García-Rodríguez, Yoon, & Secades-Villa, 2015; Yi et al., 2008; Yoon, Higgins, Bradstreet, Badger, & Thomas, 2009), one study included opioid-dependent patients (Landes, Christensen, & Bickel, 2012), and one study included marijuana-dependent individuals (Peters, Petry, LaPaglia, Reynolds, & Carroll, 2013). One study (16.5%) found expected reductions in DD, one found mixed results on two different DD tasks (16.5%), and four found null results or unexpected increases in DD (67%). Regarding behavioral outcomes, four studies (67%) found significant reductions in smoking behavior. None of the studies tested whether decreases in substance use were mediated by decreases in DD rates.

The four studies that included smokers or treatment-seeking smokers examined effects on both DD and smoking behavior. Yi et al. (2008) included two DD tasks—one employing monetary rewards and one employing cigarette rewards. They found that monetary as well as cigarette DD was decreased in the CM condition, whereas no changes were found in the control condition. However, the CM condition was not directly compared with the control condition in the statistical analyses. Furthermore, a decrease in carbon monoxide (CO) levels, measuring how much carbon monoxide is present in the exhaled air of the smoker as a proxy of smoking behavior (Deveci, Deveci, Açik, & Ozan, 2004) over time was found in the CM condition. Yet no results on CO levels were provided for the control condition, which did not allow a direct comparison between both conditions.

Yoon et al. (2009) performed two DD tasks, one including monetary rewards available immediately and after a delay. The other task included cigarette rewards available immediately and monetary rewards available after a delay. They found attenuated DD in the CM condition on the task comparing monetary and cigarette rewards, but no effects on the monetary DD task. Moreover, participants in the CM condition had lower CO levels posttraining than participants in the control condition did. Weidberg et al. (2015) did not find direct effects of CM on DD, but they demonstrated an increase in smoking abstinence in the CM condition at posttest though not at follow-up. In a study by Kurti and Dallery (2014), CM and exercise were tested in several combinations to see what effects could be found on DD. Neither exercise nor CM decreased DD rates. However, the conditions including CM found increased latencies to smoke and decreased total puffs in smokers compared with conditions without CM.

Landes et al. (2012) found that DD decreased after a CM training in opioid-dependent patients; however, the control condition that only received buprenorphine also showed attenuated levels of DD. Peters et al. (2013) compared a CBT-only condition with three conditions including CM in marijuana-dependent individuals and found increases in DD in the CBT-only group, whereas DD rates remained stable in the CM conditions. Both studies did not find effects on marijuana (Peters et al., 2013) and opioid use (Landes et al., 2012).

In summary, findings regarding CM and its ability to decrease DD are mixed. Studies that included a substance-specific DD task (Yi et al., 2008; Yoon et al., 2009) yielded more positive effects than studies that used monetary DD tasks. The two studies with expected or mixed effects included a substance-specific DD task. These latter findings, in combination with the promising effects of CM on behavior (67%), could be an indication that the effects of CM are substance specific and do not transfer to monetary DD tasks. More research is needed to confirm whether DD functions as a mediator that reduces substance use by CM or whether other mechanisms are at work to explain the behavioral effects.

#### Money-management-based training

Two studies exposed their participants to some sort of money-management-based training, based on the idea that more knowledge of money management increases the salience of future rewards and makes those more concrete. One study found null results for DD, but the expected results for cocaine abstinence; the other study found the expected reductions in DD. Black and Rosen (2011) allocated their participants either to the advisor-teller money manager (ATM) training condition—which is a multicomponent training that includes substance abuse treatment in the context of discussions on other money-management concerns—or to a control condition. Patients in the ATM condition were stimulated to create monthly budgets that reflect long-term goals, broken down into short-term spending plans. The authors found that discounting rates increased and cocaine abstinence rates decreased in the control condition and both discounting rates and cocaine abstinence remained stable in the ATM condition. DeHart and colleagues (DeHart, Friedel, Lown, & Odum, 2016) tested college students that were either following a personal finance course focused on basic financial education or an abnormal psychology course. DD decreased in the financial education condition at the end of the semester, whereas there was no change found in DD rates in the abnormal psychology condition.

In summary, the two studies provide mixed evidence for decreases in DD as a function of money-management-based trainings. More research is needed to get a better understanding of the underlying mechanisms driving this effect and the relevance of this effect in clinical populations.

#### Brief motivational training in combination with substance-free activity session

Brief motivational trainings are often used as trainings for substance-use problems, by including substance-related risks, personalized feedback about substance-use patterns, and harm-reduction strategies using a motivational interviewing style (Miller & Rollnick, 2002). In the two studies described here, a brief motivational training was combined with a substance-free activity session (SFAS) and compared with either an education session as a control condition (Dennhardt, Yurasek, & Murphy, 2015) or a relaxation training as a control condition (Murphy et al., 2012). SFAS was delivered to heighten engagement in substance-free alternative activities, increase the importance of academic and career goals, and draw attention to the negative link between substance use and reaching goals. Both studies did not find any effects on DD. However, Murphy and colleagues found decreased self-reports of alcohol problems in the condition that received the brief motivational training in combination with SFAS. In summary, brief motivational training in combination with SFAS do not seem to be effective in reducing DD rates. Yet there is tentative evidence suggesting that they could reduce alcohol problems.

#### Cognitive behavioral therapy

The group-based addiction cognitive behavioral therapy (CBT) programs employed in the two experiments by De Wilde, Bechara, Sabbe, Hulstijn, and Dom (2013) in polysubstance dependent alcoholics and the study by Secades-Villa and colleagues (Secades-Villa, Weidberg, García-Rodríguez, Fernández-Hermida, & Yoon, 2014) in treatment0seeking smokers included among other components psychoeducation, self-monitoring, coping-skills training, stress management, and relapse-prevention strategies. Both studies did not find effects of CBT on DD at posttest. Secades-Villa et al. (2014) found a difference between smokers and abstainers on DD on 12-month follow-up, suggesting that DD rates were decreased for abstainers compared with smokers, although they did not include a control condition. Both studies did not include measurements of smoking behavior to test for changes in smoking behavior over time. In summary, there is currently no evidence supporting that CBT reduces DD.

#### Acceptance-based/mindfulness-based trainings

Most studies hypothesize that participants with heightened DD demonstrate an increased focus on immediate rewards and attempt to train participants away from the present moment to a future-orientated state. Others hypothesized that participants with heightened DD focus on the aversive properties of waiting and therefore should be trained to concentrate on the present moment and accept all feelings experienced at that moment. For example, if aversive events such as craving are intolerable, then a smoker will escape or avoid these events by smoking (i.e., choosing for the immediate reward). Acceptance-based and mindfulness-based strategies endorse an individual’s willingness to experience what cannot be controlled, such as stress or craving, and support choices that are grounded in awareness of the present moment (Zettle, 2007).

Four studies included an acceptance-based or mindfulness-based approach, two of which found significant decreases in DD and two of which found mixed results. In a study by Morrison and colleagues (Morrison, Madden, Odum, Friedel, and Twohig, 2014), a brief acceptance-based training, designed to increase willingness to experience discomfort, successfully decreased DD. Yao et al. (2017) compared a healthy control sample with a sample of individuals with Internet gaming disorder (IGD) and found that discounting rates and IGD symptoms decreased in the IGD group after an acceptance-based training. Yet, at baseline, the IGD group already showed higher DD rates than the healthy control group, and no active control condition was included. These two factors make it difficult to fully interpret the findings, since decreases in DD rates can also be caused by other factors than the training itself, such as learning effects, regression to the mean, or expectations (Boot, Simons, Stothart, & Stutts, 2013; Cohen, Cohen, West, & Aiken, 2013). In addition, in two studies by Hendrickson and Rasmussen (2013, 2017), a mindfulness training was tested to examine the effects on impulsive choice patterns for food and money. The participants in the mindfulness training condition showed attenuated discounting rates for food, but not for money, compared with the control condition. Although the number of studies is limited, acceptance-based or mindfulness-based strategies seem to be a promising avenue to be continued in future research.

#### Working memory training

Working memory (WM) is a core component of executive functioning and is defined as the ability to briefly hold in mind and manipulate small amounts of information to use in the execution of cognitive tasks (Cowan, 2014). WM training constitutes, for example, memorizing a sequence of numbers in the original and reverse order or recognizing a list of words out of bigger list of words. Research has demonstrated significant correlations between measures of WM and DD (Bobova et al., 2009; Shamosh et al., 2008). These correlations are substantiated by brain areas of functional overlap during tasks of WM and DD (Wesley & Bickel, 2014). Improving WM could therefore possibly also decrease discounting rates. Two studies employed a WM training to reduce DD, one with results in the expected direction on DD whereas the other study did not find any effects.

In the study by Bickel et al. (2011b) in stimulant-dependent individuals during substance-abuse treatment, decreased discounting rates were found for participants in the WM training condition compared with the control condition. In contrast, Rass et al. (2015) applied almost the same WM training and did not find a difference in DD rates between the WM and control condition. Though the content of the WM training in both studies was almost the same, several other differences between studies could have caused the contradicting results. Each study included different substance-addicted population in different addiction stages (stimulant-dependent vs. methadone-maintenance patients), and the studies also differed in training dosage (Bickel et al., b: 4–15 training sessions vs. Rass et al., 2015: 25 training sessions). Furthermore, the study by Bickel et al. (2011b) included 74% males, whereas the study by Rass et al. (2015) included 46% males. In conclusion, it still has to be discovered whether WM training is effective in decreasing DD rates and what factors could possibly affect training effects.

#### Visualization training

Some theoretical models of discounting hypothesize that delayed outcomes are less concrete or less vivid than immediate outcomes, and therefore individuals tend to prefer immediate outcomes over delayed ones (Rick & Loewenstein, 2008; Trope & Liberman, 2003). Increasing the ability to vividly imagine future events by visualization training could then theoretically lead to a decrease in discounting rates. Yet, the study by Parthasarathi and colleagues (Parthasarathi, McConnell, Luery, & Kable, 2017) employed a visualization training and did not find the expected effects on DD. In a 4-week training study, participants in the visualization condition received 1-hour guided meditation sessions followed by goal-oriented guided visualization. Participants had to focus on a future-oriented goal and had to vividly imagine scenarios overcoming the obstacles in their way and experiencing the feelings associated with achieving the goal. The control group received guided relaxation, without visualization or future thinking. Based on this one study, it still has to be seen whether visualization training is effective in decreasing DD (however, see Episodic Future Thinking results below).

### Manipulations

#### Future

##### Episodic future thinking

Episodic future thinking (EFT) is the ability to vividly imagine the future (Benoit, Gilbert, & Burgess, 2011) and thereby to preexperience future events (Atance & O’Neill, 2001). Although small differences between experiments exist, most EFT manipulations ask participants to first compile a list of upcoming events (e.g., wedding, party, vacation) at several moments in time in the future and rate them on variables such as personal relevance, valence, and arousal (Peters & Büchel, 2010). Thereafter, participants need to perform a different version of the DD task in which the amounts of money in the larger future reward were paired with a subject-specific verbal episodic tag indicating to the participants which event they had planned at the respective day of reward delivery (Peters & Büchel, 2010). The content of control conditions differed between studies; for example, some studies asked participants to complete a standard DD task, whereas others instructed their participants to imagine recent events instead of future events. Twenty out of the 24 experiments (83%) employing an EFT manipulation found the expected reductions in DD in the EFT manipulation compared with the control condition. Five studies (Chiou & Wu, 2016; Daniel et al., 2013b; Dassen et al., 2016; Snider, LaConte, & Bickel, 2015; Stein et al., 2016) also tested for effects on behavior and found reductions in smoking, caloric intake, and hypothetical alcohol purchase. For example, in the study by Daniel et al. (2013b) they found that the participants in the EFT condition showed lower DD rates and lower caloric intake, compared with the control condition.

Three experiments (13%) did not find that EFT reduced DD and one experiment (4%) found mixed results (i.e., DD decreased in healthy control sample after EFT, but not in the amnestic patients’ sample). In Experiment 2 of the study by L. Liu, Feng, Chen, and Li (2013), a negative future thinking manipulation (i.e., EFT manipulation with negative events to be imagined) was tested against a control condition. DD rates increased in the negative EFT condition. In Experiment 3, a neutral future thinking manipulation (i.e., EFT manipulation with neutral events to be imagined) was tested against a control condition, and no differences were found between both conditions. The authors concluded that the valence of the imagined future reward matters for the effectiveness of the manipulation, since a decrease in DD rates was found in Experiment 1, where a positive future thinking manipulation (i.e., EFT manipulation with positive events to be imagined) was employed. In Experiment 1 of the study by Palombo and colleagues (Palombo, Keane, & Verfaellie, 2016), amnestic and healthy control subjects performed either a standard DD task (control condition) or a DD task with EFT manipulation. EFT reduced DD in healthy control subjects, but not in amnestic subjects.

In conclusion, EFT manipulations are one of the more thoroughly researched categories, and the results on DD and behavior are mostly positive and promising. Nevertheless, the few studies that reported null findings suggest that EFT needs to be positive in valence, and individuals’ episodic memory needs to be intact for the manipulation to be effective.

##### Connectivity to future (self)

Manipulating one’s connectedness to the future (self) could lead to more patient behavior, based on the notion that a higher connectedness to the future (self) implies a greater willingness to defer benefits to the future self. Eleven experiments tested manipulations to heighten connectivity to the future (self) and 10 (91%) were effective in reducing DD rates. Bartels and Urminsky (2011) employed multiple experiments and discovered that directly informing people that identity is constant over time and implicitly inducing the opinion that personality stays the same over time reduced DD. Kuo, Lee, and Chiou (2016) showed participants with the intention to lose weight either a weight-reduced “ideal” self or their present self in a virtual reality environment. Compared with the control group, participants who viewed their weight-reduced avatars showed attenuated DD. Furthermore, participants in the weight-reduced condition also ate less ice cream in a taste test and were more likely to choose a sugar-free drink as a reward, and this effect was mediated by discounting rate.

In an experiment by Sheffer et al. (2016), participants were exposed to a set of words with either a present focus (PF; i.e., instant), future focus (FF; i.e., self-control), or nontemporal focus (NTF; i.e., pale). Participants in the FF condition exhibited significantly lower discounting rates than those in the PF or NTF conditions. Israel, Rosenboim, and Shavit (2014) tested a priming manipulation, where they expected attenuated DD when participants were primed with older people (as a proxy for their own older selves). Participants either saw pictures of a vacation, older people, or were not exposed to any prime. The older people prime resulted in attenuated DD, whereas the vacation prime resulted in heightened DD. Pronin and colleagues (Pronin, Olivola, & Kennedy, 2008) found that choosing for your future self attenuated DD in comparison with choosing for the self.

In two experiments by Joshi and Fast (2013), participants were manipulated by giving them the role of a manager, or they had to think about an event where they had power over somebody else. The authors hypothesize that people with high power report higher connectedness to their future selves as compared with people with less power, and therefore show lower DD rates. Both experiments confirmed this hypothesis by significant differences between the high and low power conditions, with participants in the high power condition showing lower DD rates.

One experiment (9%) showed a trend toward a decrease in DD (*p* = .056), but did not convincingly show effects of the connection to future (self) manipulation on DD. In the experiment by Hershfield et al. (2011), participants in the experimental condition saw an avatar of their future self in a virtual-reality environment, whereas in the control condition participants saw an aged avatar of someone else.

In summary, connectivity to future (self) manipulations can be promising in reducing DD rates. However, more research into the best method and doses to create a connection to the future (self) is needed to support these initial findings.

##### Construal level manipulation

Construal level theory (CLT) proposes that temporal distance alters people’s responses to future events by systematically changing the way people mentally represent those events (Trope & Liberman, 2003). When temporal distances are larger, events are more likely to be represented in abstract terms that capture their central features (high-level construals) than in more concrete and incidental terms (low-level construals). The studies by Kelley and Schmeichel (2015), Li et al. (2016), and Malkoc, Zauberman, and Bettman (2010) tested the hypothesis that higher-level construals could lead to more patient behavior. This hypothesis is grounded in the argument that if a bias to the present is driven by contextual and concrete representations, then endorsing an abstract mindset should decrease the extent of these representations and thus attenuate delay discounting (Malkoc et al., 2010). All manipulations used in these studies tried to induce a higher-level construal by enhancing an abstract mindset—for example, by semantic priming of abstract and concrete words (Malkoc et al., 2010). All seven experiments found the expected effects, meaning that more abstract mindsets attenuated DD.

In contrast to the abovementioned experiments, H. Kim, Schnall, and White (2013; Studies 1a–b, 2) tested the hypothesis that lower-level construals could lead to more patient behavior. The rationale behind this hypothesis is that the more detailed information is available, the more a future event can be construed in a concrete way, corresponding to a low-level construal. In all three experiments, participants were willing to wait longer as they were offered a trip to Paris with the value of the monetary reward, instead of a monetary reward itself. As more details were given about the trip, such as sightseeing possibilities and accommodations, the effect was even stronger, suggesting that a more specified event increases patience. The effects found in the experiments of H. Kim et al. (2013) come close to those found in the EFT and connectivity to future (self) experiments. Different theoretical frameworks and methods are used, but in the end the future event is made available and more concrete to the participant.

In addition to temporal distance, spatial distance can also influence subjective judgment of future time, an instance of metaphoric transfer (B. K. Kim, Zauberman, & Bettman, 2012). According to this theory, spatial distance will influence how long or short individuals judge a future time to be, when spatial distance information is available in a temporal judgment context and is associated with the judgment of future time. For example, an individual in Los Angeles may perceive the same 6-month duration from today to be longer when she is expecting to be in New York 6 months later than when she is expecting to be in San Francisco. In two experiments by B. K. Kim et al. (2012), spatial distance was manipulated by instructing participants to either mentally imagine two locations far away from each other or close to each other. Short spatial distance manipulation reduced DD, thus a smaller spatial distance between two locations leads to less delay discounting in intertemporal choices (B. K. Kim et al., 2012).

In conclusion, both abstract and concrete mindsets, including mentally imagining a smaller spatial distance, seem to be effective in decreasing DD rates. The findings regarding the concrete mindset fit the findings of EFT and connectivity to future (self) manipulations.

#### Social factors

##### Social context

The presence of a social context could influence decision-making, based on the rationale that when an individual makes a choice on behalf of their group (i.e., including themselves), they prefer alternatives geared towards the group’s long-term benefit (Bickel, Jarmolowicz, Mueller, Franck, et al., 2012a). Five experiments tested effects of the presence of a social context on DD; 80% (*n* = 4) found positive effects on DD, whereas 20% (*n* = 1) did not find main effects of condition on DD. Experiments that found positive effects were conducted by Charlton et al. (2013). In their DD task, participants had to make choices for themselves and for a group of people, with the amount of money that an individual could earn being constant. Situations where people had to make choices for themselves were related to less patient choices than situations where people made decisions for the whole group. In comparison, Yi et al. (2008) employed the same manipulation, but did not find a positive effect of choosing for the group over choosing for the self. Finally, Bickel, Jarmolowicz, Mueller, Franck, and colleagues (2012a) tested a combination of two manipulations—namely, choosing for a present versus future self and choosing for me versus we. They found that choosing for multiple people (“we condition”) was most important in bringing down DD rates. In conclusion, social context manipulations seem to reduce DD.

##### Social influence

Three experiments by Senecal and colleagues (Senecal, Wang, Thompson, & Kable, 2012) tested the effect of normative strategies (i.e., individuals should compare each intertemporal choice against the other investment and borrowing options available to them) and social influence by peers on DD. When information about normative strategy for economic decision-making was made available to participants, DD rates decreased, although no direct comparison between conditions was made in Experiment 1 and no control condition was used in Study 2. Furthermore, peer-generated advice about strategies for economic decisions only decreased DD when this advice was written down in a patient versus impatient way (yet see our discussion of experimenter-demand effects in the discussion). Based on this one study, social influence manipulations seem to reduce DD, but more research is needed.

##### Emotion

Emotion is multidimensional and can influence many cognitions, including attention, sensory perception, and memory. Twenty-six experiments all used very different forms of inducing affect or emotional priming with the aim to decrease DD, and 22 (85%) found a decrease in DD rates. Guan, Cheng, Fan, and Li (2015) showed their participants positive, neutral, and negative pictures and then asked them to perform a DD task. They found that DD was attenuated in the neutral condition and even more decreased in the positive condition, compared with the negative condition. Ifcher and Zarghamee (2011) used short movie clips, and Pyone and Isen (2011) used words to induce positive or neutral affect, and both found that positive affect reduced DD. In contrast, Augustine and Larsen (2011) and Hirsh, Guindon, Morisano, and Peterson (2010) did not find a reduction in DD rates while using the same sort of affect induction.

In five experiments by Berry and colleagues (Berry, Sweeney, Morath, Odum, & Jordan, 2014; Berry et al., 2015) and van der Wal and colleagues (van der Wal, Schade, Krabbendam, & Van Vugt, 2013), participants were exposed to natural versus built environments (and geometric shapes in the Berry et al., 2014, study). Participants in the natural environment manipulation showed attenuated DD rates compared with participants in the urban environment manipulation. There are several possible explanations for these findings. For example, exposure to natural environments decreases stress, increases happiness, improves mood, and restores attention (Berto, 2005; Bowler, Buyung-Ali, Knight, & Pullin, 2010; White, Alcock, Wheeler, & Depledge, 2013). Natural environments are serene, thereby increasing attentional capacity and/or reducing general arousal, and by viewing them perception of time is lengthened (Berry et al., 2014; Berry et al., 2015). Another, evolutionary, explanation states that natural environments indicate resource abundance, and therefore individuals choose more often for larger but future outcomes (van der Wal et al., 2013). Built environments may indicate competition for resources and mates, and therefore individuals more often choose for smaller but immediate outcomes.

In the experiments by DeSteno, Dickens, and Lerner (2014) and Dickens and Desteno (2016), a gratitude manipulation was used, and both experiments found that this manipulation led to lower discounting rates, although the experiment by Dickens and Desteno (2016) did not include a proper control condition. In two experiments by Huang, Huang, and Wyer (2016), a nostalgia manipulation (i.e., reminiscing about positive events in the past) was used and participants in the nostalgia conditions showed decreased DD rates compared with the control condition. In the experiment by Raeva and colleagues (Raeva, Mittone, & Schwarzbach, 2010), both regret and rejoicing were induced. Results suggested that when regret was experienced, participants preferred the immediate rewards, whereas when rejoicing was experienced, participants chose the delayed reward more often.

Berndsen and van der Pligt (2001) used an optimism manipulation and found that participants in the low-optimism condition showed decreased discounting rates compared with the high-optimism condition. In addition, Luo, Ainslie, and Monterosso (2014) induced fear, happiness, and neutrality by using facial expressions whereby fear decreased DD rates compared with the happiness and neutrality conditions. Callan, Harvey, and Sutton (2014) demonstrated in two experiments that a manipulation regarding the derogation of victims of misfortune, although damaging to others, yielded a psychological benefit for the self by choosing more often for the larger–later rewards than for the smaller–sooner rewards. W. Liu and Aaker (2007) proposed that an experience of the death of someone close prompts people to notice and reflect upon their long-term futures, causing changes in their intertemporal decisions. In one of their experiments, they showed that besides the actual experience of a cancer death, mental simulation of experiencing cancer death leads to decreases in DD rates as well. This effect could be interpreted as increased salience and concreteness regarding one’s future life course, shifting focus away from the present toward the long run, hence fitting nicely with the studies on EFT and making the future more concrete. The experiment of Quisenberry and colleagues (Quisenberry, Eddy, Patterson, Franck, & Bickel, 2015) on delay to sexual gratification employed three conditions: a positive health consequence condition, a negative health consequence condition, or a negative health consequence with the expression of regret condition. No effects were found on DD.

To summarize, most experiments (85%) in the emotion priming category found positive results on DD. Sixteen experiments induced positive affect or primed positive emotions and thereby found decreased DD rates, whereas six other experiments induced negative affect or primed negative emotions and found decreased DD rates. Thus, both positive and negative affect/emotional priming can result in decreases in DD; yet there were also some studies, with both positive and negative affect/emotion inductions, that did not find effects in the expected direction (15%). These inconsistent findings leave the exact interaction between emotion and DD to be uncovered.

#### Framing

##### Bundling

Bundling refers to aggregating choices between temporally extended series of smaller–sooner/larger–later alternatives rather than case-by-case choices (Ainslie, 2001). Four experiments have employed a bundling manipulation, of which three (75%) found positive results on DD and one (25%) did not. In the experiments by Hofmeyr, Ainsli, Charlton, and Ross (2011) and Kirby and Guastello (2001), participants were allocated to either a free, suggested, or forced condition. In the free condition, participants could choose between a smaller–sooner reward and a larger–later reward at several consecutive moments (trials) over time (one choice today, one choice in 2 weeks, one choice in 4 weeks, etc.). In the suggested condition, participants were offered the same choices as in the free condition at several moments over time; however, accompanying the first choice, it was suggested to participants that their choice made in this first trial most of the time predicts their choices in the future. After this, participants were still free to make different choices in the later trials than in the first trial. In the forced condition, participants had to choose in the first trial for the rest of all trials, thus for a set of rewards. In both studies, discounting rates decreased when participants were allocated to the suggested condition and even more in the forced condition. Choosing for a set of rewards in the future instead of making those choices one by one thus seems to decrease DD rates. In contrast, in the study by Białaszek and Ostaszewski (2012) this effect was not found—in fact, the effect was reversed for large rewards; DD rates decreased when participants choose for a single large reward instead of a sequence of large rewards. Based on the four studies, bundling seems to decrease DD rates, but may partly depend on reward magnitude. Long-term effects and behavioral relevance need to be distinguished.

##### Time framing

Time framing manipulations make subtle changes to the time component in standard DD tasks, where most of the experiments altered the way delays were described. Twenty-one experiments attempted to decrease DD by time framing manipulations, 16 (76%) found the expected effects on DD, and five (24%) did not find effects on DD. In the experiments by DeHart and Odum (2015); Dshemuchadse, Scherbaum, and Goschke (2013); LeBoeuf (2006); Klapproth (2012); and Read and colleagues (Read, Frederick, Orsel, & Rahman, 2005), delays were either construed as temporal distances (e.g., 6 days, 2 months) or specific dates (e.g., November 21). All experiments found attenuated DD when delays were presented as specific dates instead of temporal distances. The experiment by Lempert, Johnson, and Phelps (2016) employed the same manipulation but did not replicate the findings of the previous studies. One important difference between studies that could explain this failure to replicate is that Lempert et al. (2016) used a within-subject design with randomly intermixed trials, whereas all other studies employed a between-subject design or used a within-subject design but presented the date and delay conditions in separate blocks. Since discount rates are susceptible to order presentation (Robles & Vargas, 2008), this may explain the failure to replicate the date/delay effect.

Another time framing manipulation was performed by Dai and Fishbach (2013). In this study, participants were either allocated to a near-future, distant-future, or waiting condition. In the near-future condition, participants chose between, for example, (a) $50 in 3 days and (b) $55 in 23 days. In the distant-future condition, participants chose between, for example, (a) $50 in 30 days and (b) $55 in 50 days. In the waiting condition, participants read about two choice options: (a) $50 in 30 days and (b) $55 in 50 days (similar to the distant-future condition), but they did not need to make any choice right away. After 27 days of waiting, the experimenter send the participants a note asking them to choose. At that time, participants were facing options identical to those in the near-future condition. It was found that participants in the waiting condition showed decreased DD compared with participants in both the near-future and distant-future conditions.

Of the other four experiments that did not find effects, the experiment by Zauberman and colleagues (Zauberman, Kim, Malkoc, & Bettman, 2009) manipulated participants by making duration more salient to them by having them estimate the duration of several activities. Ebert and Prelec (2007) specifically instructed their participants to focus particularly on the future arrival time of the reward they could earn. The experiment by Rabinovich and colleagues (Rabinovich, Morton, & Postmes, 2010) asked participants to think about what their financial circumstances were likely to be after 1 month (short-term time perspective condition) or after 5 years (long-term time perspective condition).

In conclusion, the majority of time framing manipulations found decreases in DD rates. Yet the long-term effects and clinical relevance need investigation.

##### Reframing of rewards

In DD tasks, participants often have to choose between two amounts of money. However, the way these two amounts are described seem to matter according to 26 experiments. Of these 26 experiments, 25 (96%) found positive results of the reframing of reward manipulation on DD. An explicit zero manipulation was employed by Magen, Dweck, and Gross (2008); Magen, Kim, Dweck, Gross, and McClure (2014); and Radu et al. (2011). Participants in the hidden zero (= control) condition chose between a smaller–sooner ($5 today) and a larger–later ($50 in 2 weeks) reward, whereas participants in the explicit zero condition chose between a smaller–sooner reward ($5 today and $0 in the future or $0 today and $50 in 2 weeks). Explicitly referring to the hidden zero in each choice alternative decreased DD rates. In three experiments by Fassbender et al. (2014), half of the trials existed of rounded decimal values (e.g., $11.00), whereas the other half of the trials were nonzero decimal values (e.g., $11.72). Discount rates decreased when intertemporal choices were constructed of monetary outcomes with nonzero decimal values.

Weatherly, Derenne, & Terrell (2010), Weatherly and Derenne (2011), and Weatherly and Terrell (2010) gave a DD task to participants in two conditions: In the “won” condition, participants were told that the amounts were money they had won; in the “owed” condition, participants were told that the amounts were money they owed (e.g., your own money that you lent to someone and need to get back). Results showed that participants had lower discounting rates when money was “owed” than when money was “won,” indicating that won money was less valued than their own money. In two experiments by Read and colleagues (Read, Frederick, & Scholten, 2013, Experiments 1 and 2), conditions including investment language in the choice alternatives (i.e., “Would you rather receive $70,000 now or invest it for 1 year at an 8% interest rate?”) decreased DD rates, whereas Experiment 3 did not replicate this effect.

Appelt, Hardisty, and Weber (2011); Grace & McLean (2005); and Weber et al. (2007) compared effects of delay versus acceleration frames on discounting rates. Participants in an acceleration frame (i.e., receiving $75 in 3 months or receiving a smaller amount now) more often chose the delayed reward than the immediate reward, than did participants in a delay frame (i.e., receiving $50 now or receiving a larger amount in 3 months). Finally, Jiang, Hu, and Zhu (2014) introduced up-front losses as well as gains; participants in the control responded to the typical choice pairs (i.e., gain $120 in a week vs. gain $150 in 4 weeks), whereas in the up-front loss or win condition, both rewards options began with a same immediate loss or win (i.e., lose [or win] $100 now and gain $120 in a week vs. lose [or win] $100 now and gain $150 in 4 weeks). The addition of both up-front losses and gains in the choice pairs reduced DD.

In sum, manipulations that reframe rewards, such as explicitly referring to the hidden zero in choices and including nonzero decimal values, seem to be highly effective in decreasing DD rates. Yet, again, the long-term and clinical relevance effects need to be investigated.

## Discussion

The current systematic review provides an overview of all studies that have attempted to decrease DD by means of behavioral trainings or manipulations. In this review, 98 studies were discussed regarding their effectiveness in reducing DD, but also in changing real-life behavior if measured.

### Overall effectiveness of trainings and manipulations

Generally, the majority of published studies (*n* = 119; 79%) were able to reduce DD, indicating that although there may be between-subject stability to DD, it is also profoundly context dependent within individuals. Of the 132 manipulation experiments, 114 (86%) were able to decrease DD, whereas from the 19 trainings, only five (26%) were able to decrease DD. One would expect that a more thorough, longer lasting, and active training would be more effective than a short manipulation, especially since most trainings have been performed in populations with initially steep discount rates, leaving room for rate dependence and regression to the mean (Bickel, Quisenberry, and Snider, 2016a). Yet we found the opposite, and there are multiple reasons to explain this finding.

First, the manipulation studies more often employed a within-subject design (*n* = 38, 29%) compared with training studies (*n* = 4, 21%). Within-subject designs have more power to detect effects than between-subject designs do (Charness, Gneezy, & Kuhn, 2012). Second, the majority of trainings tried to find effects over longer time periods, while the effects of most manipulations were tested just after the manipulation. We know that training effects tend to decay over time, with effect sizes generally being the largest at posttest and decreasing at follow-up (Cuijpers, van Straten, Smit, Mihalopoulos, & Beekman, 2008; Moyer, Finney, Swearingen, & Vergun, 2002; Prendergast, Podus, Finney, Greenwell, & Roll, 2006; Wilfley et al., 2007).

Third, trainings were mostly conducted in clinical populations and the manipulations (with a few exceptions) in healthy populations. Attrition in clinical studies is common and is frequently seen in studies with substance-abuse samples (Brorson, Arnevik, Rand-Hendriksen, & Duckert, 2013). Attrition in the training studies reported in this systematic review was on average 15% (range: 0%–50%), with higher dropout rates in more severely affected populations. Following intention-to-treat principles, all participants should be included in analyses, regardless of whether they actually received treatment, and thus dropouts are included in the analyses as nonabstainers (Brorson et al., 2013; Montori and Guyatt, 2001). Hence, comparing manipulations in healthy control samples with a higher chance of success with trainings in clinical samples can lead to biased interpretations.

Finally, publication bias could have introduced an overestimation of the actual effect of trainings and manipulations on DD (Hopewell, Clarke, Stewart, & Tierney, 2007; Mervis, 2014). In the current systematic review, the publication rate of null results of training studies (74%) was higher than the publication rate of null results of manipulation studies (14%). It is likely that publication bias is more prevalent in the manipulation studies, given that these studies are less complex to perform compared with training studies. With the scientific field moving forward, transparency and replicability have become increasingly important. Future well-designed studies in the field will likely adhere to the three core practices of disclosure, registration, and preanalysis plans, as well as providing open access to data and material, and will go a long way toward addressing this file-drawer problem (Miguel et al., 2014; Simmons, Nelson, & Simonsohn, 2011).

### Implications: Most promising strategies to reduce DD

#### Trainings

Based on the results of the training studies, the acceptance-based/mindfulness-based trainings seem to be most promising in reducing DD. The precise mechanisms by which these mindfulness-based trainings work, however, are still unclear. Most intervention theories related to elevated DD rates attempt to change individuals’ heightened emphasis on immediate rewards toward a more future-oriented focus, whereas acceptance-based/mindfulness-based trainings concentrate on experiencing and accepting the aversiveness of the present moment (Ashe, Newman, & Wilson, 2015). This latter view provides another lens on impulsivity in general: more impulsive people might find waiting in the moment particularly aversive, and their prime motivation is to escape those negative feelings by choosing immediate rewards (see delay aversion theory by Sonuga-Barke, 2005). Training them to mindfully attend to the present can help them to get through these moments and choose for the future reward. Considering the transference of effects to behavior, one might, for example, think that through acceptance-based/mindfulness-based trainings, individuals with addictive problems learn to accept negative feelings associated with moments of cravings and therefore get through these moments and stay abstinent (Ashe et al., 2015). Indeed, this alternative focus on improving DD by decreasing the aversiveness of the present moment is strengthened with significant effects on behavior, such as food behaviors (for review, see Olson & Emery, 2015), addictive behaviors (e.g., Gifford et al., 2004; Petersen & Zettle, 2009), and other mental and physical health problems (for review, see A-Tjak et al., 2015).

Although the effects regarding CM on DD rates in this systematic review are mixed (only 33.3% found the expected decreases in DD), most of these studies (66.6%) found positive effects on health outcomes. This is further supported by a high number of studies in the literature that directly measured effects of CM on health outcomes in clinical samples (and not measuring DD rates). Multiple systematic reviews and meta-analyses show that CM has beneficial effects on abstinence rates in a range of substance-abuse samples (Dutra et al., 2008; Giles, Robalino, McColl, Sniehotta, & Adams, 2014; Griffith, Rowan-Szal, Roark, & Simpson, 2000; Lussier, Heil, Mongeon, Badger, & Higgins, 2006; Prendergast et al., 2006; Schumacher et al., 2007). However, it remains unclear whether changes in behavior are mediated by changes in DD and, if so, whether these effects are substance specific (Stanger et al., 2013). Future research should test this mediation hypothesis and, if necessary, come up with alternative explanations for the effects of CM on behavior.

#### Manipulations

The majority of the manipulations (86%) seem to significantly reduce DD rates. However, decades of intervention research have shown that health behaviors or substance abuse are not easily changeable (Jeffery, 2004). In addition, the effects of the manipulations could very well be short-lived and context-specific effects that quickly disappear over time. For instance, framing effects might be useful as a “nudge” if the choice environment can be controlled, but perhaps less useful for bringing about durable person-level change. Therefore, the field would benefit from identifying (with tightly controlled experiments) the most promising manipulations; these manipulations should be informed by theoretical models that link predicted changes in DD as well as in behavior, ultimately.

With those recommendations in mind, the most encouraging line of research regarding manipulations seems to be the “future” category, especially “future episodic thinking” and “connectivity to future (self)” manipulations with 83% and 91% effectiveness rates, respectively. These studies are grounded in solid theory, find robust effects on DD rates, and the EFT studies include healthy (62%) as well as different clinical samples (38%) and find promising effects on smoking behavior (40%), caloric intake (40%) and hypothetical alcohol purchase (20%). Thus, while many studies show that manipulations involving a future orientation reduce DD rates and have promising effects on a variety of health behaviors, recent research has started to evaluate alternative driving factors of these effects, such as demand characteristics. In a study specifically focusing on EFT, Rung and Madden (2018a) state that demand characteristics will probably be inherent to EFT procedures, as participants are asked to create future thinking cues that in turn are embedded in the DD tasks they have to perform later on. Being aware of the hypothesized effects in a study may bias participants’ answers and behaviors, potentially driving the effect of EFT on DD and behavior (Rung & Madden, 2018a). Besides effects on EFT, demand characteristics could likely play a role in “connectivity to future (self)” manipulations and “construal level thinking manipulations (concretization).”

To optimize these effects, it is recommended to extend these “future” manipulations into longer, multiple session trainings and use rigorous transparent designs to test effects in the long term in adequately powered samples (Boot et al., 2013; Miguel et al., 2014; Simmons et al., 2011). These recommendations could be elegantly implemented by the use of technological tools. In particular, video games are a ubiquitous part of our current society (Granic, Lobel, & Engels, 2014) and are able to evoke intrinsic motivation to engage people in the treatment process (Ryan, Rigby, & Przybylski, 2006). Moreover, video games can promote long-term training by incorporating repetitive actions and encouraging repetitive gameplay without evoking boredom (Granic et al., 2014; C. S. Green & Bavalier, 2012). Finally, to illustrate its promise and clinical relevance, some of these manipulations have already been included in trainings with relevant clinical populations: Solanto (2011) developed a CBT program for adults with ADHD focusing on executive functions and integrated the visualization of long-term rewards and positive outcomes (episodic future thinking) as an important part of this effective intervention.

### Overarching mechanisms of change

Although the content of the trainings and manipulations differed broadly between studies, there are some overarching mechanisms of change that could explain results over different trainings and manipulations, content wise. Please bear in mind that we have subjectively divided all studies into different categories, for structuring and clarification purposes. However, it could well be that multiple categories of trainings and manipulations originated from the same theoretical framework.

First, the perception of time within individuals is an important factor influencing DD rates (Baumann & Odum, 2012). Killeen (2015) defines impulsivity as greater control by events close in psychological time and space than by those more distal: Attention is captured by the now more eagerly than by the when. Indeed, individuals showing steeper discounting also show a more present orientation and a shorter future time perspective, which is associated with a range of problematic behaviors (Teuscher & Mitchell, 2011). For example, individuals with ADHD symptoms show a larger present orientation and a more negative view of the future than the controls (Carelli & Wiberg, 2012). Furthermore, present time perspective is significantly correlated with risky driving (Zimbardo, Keough, & Boyd, 1997) and substance use (Wills, Sandy, & Yaeger, 2001). Thus, shifting individuals’ time perception by making future rewards appear closer, more concrete, or easier to imagine might decrease DD rates. The perceived closeness of future rewards can be manipulated in different ways, such as by changing the way future delays (time framing manipulations) or rewards (reframing of reward manipulations) are perceived, by providing a richer and salient context for the future reward (EFT, construal level: concretization manipulations) or making the future easier to imagine (connection to future [self] manipulations, visualization training).

Yet Sonuga-Barke and colleagues (Sonuga-Barke, 2005; Sonuga-Barke, Taylor, Sembi, & Smith, 1992) have hypothesized that choosing for immediate rewards, and thus steep discounting, is the result of a high aversion to delay. Especially in the ADHD field there has been an emphasis on this theoretical account, and it has been shown that individuals with ADHD experience relatively strong negative emotions during waiting times, resulting in a preference to escape delay (Mies, Ma, De Water, Buitelaar, & Scheres, 2018; Scheres, Tontsch, & Thoeny, 2013b; Van Dessel et al., 2018). Acceptance-based/mindfulness-based trainings that train individuals to mindfully attend to the present can help them to get through these moments and choose for the future reward.

Second, affective and emotional states can change DD rates, but likely depend on the specific properties of the affective state (see emotion manipulations for decreases in DD for both negative and positively valenced primes). Making the delayed reward more rewarding or emotionally salient could be the driving force behind these effects (EFT, emotion, reframing of rewards, and time framing manipulations).

Finally, another overarching mechanism driving many effects is attention. Delay is often considered as a negative characteristic of the future, whereas the magnitude of the future reward is considered as positive, since it often exceeds the value of the immediate reward. Drawing attention toward magnitude and away from delay should lead to decreased DD rates (Lempert & Phelps, 2016). Changing the way reward magnitudes are presented (reframing of reward manipulations; decimal effects) or drawing the attention to the “costs” of the immediate reward (reframing of reward manipulations; explicit-zero effects; CM trainings; money-management-based trainings) are ways to change discount rates.

Taking all this together could imply that different strategies may reduce DD by working on different components of the DD process. For some individuals, for whom delay aversion is the main reason for steep DD, acceptance-based/mindfulness-based trainings may be more appropriate, whereas for others, who are driven by reward immediacy in steep DD, the future manipulations may be more effective. These overarching mechanisms of change might become building blocks for DD theories focused on altering this trans-disease mechanism, thereby building a very strong multicomponent intervention where different mechanisms may actually complement each other.

Both thorough, longer lasting, and active trainings as well as more short lived on–off manipulations could be successful in changing DD rates and behavior, depending on the purpose of a certain intervention. One might expect that trainings are better suitable in shifting “trait” discount rates than manipulations, thereby targeting steeper baseline discount rates in more clinical samples. Yet it might also be the case that it is rather difficult to change “trait” discount rates, which makes on–off manipulations (i.e., state manipulations) more interesting to change DD rates in the moment a “critical” time points (i.e., at moments when you are deciding whether to save money for retirement or to eat a piece of pie or not). We do not have the empirical evidence to back up these speculations, and thus more research investigating longer-term effects of trainings on DD and behavior, as well as the effect of frequent “in the moment” manipulations on DD and behavior, would be very helpful for future intervention design.

### Limitations and recommendations

The widespread variability in trainings and manipulations (e.g., working memory training, future-based manipulations, framing manipulations) complicates comparison between studies. Furthermore, given the large heterogeneity between studies and the nature of this systematic review, we could not formally check for publication bias. A method to differentiate between effective studies and make comparisons more objectively, is to compute a measure of effect size (e.g., Cohen’s *d*; Cohen, 1988). Unfortunately, the vast majority of the included studies did not report effect sizes or give statistics that would allow us to calculate effect sizes. Future work could build on the current review by having a more specific focus on content (e.g., only including “future-based” manipulations) and apply meta-analytic methods to dive into more specific questions regarding the effectiveness of the included studies.

As a first recommendation, we would advise to adopt a structured procedure to operationalize and test predictions regarding reductions in DD rates. An important initial step is to figure out whether certain trainings and manipulations are able to decrease DD in healthy populations with heightened DD rates to create proof of concept for DD as a mechanism that is changeable. By testing the training or manipulation first in a healthy sample with a broad range of DD rates (from very low to very high), we can contrast these different groups and control for ceiling and floor effects. By doing this, we circumvent high costs and effort and we can exclude noneffective trainings and manipulations early in the process. When effects are established in healthy populations, the training or manipulation could be introduced to a clinical sample. Though high DD rates are associated with unhealthy behaviors, it could still be possible that decreasing DD rates does not translate into a reduction of unhealthy behaviors. In other words, at this point we do not know whether this relation between DD rates and health behaviors is correlational or causal in nature; the mere fact that discounting is correlated with psychopathology does not necessarily mean that reducing discounting would reduce psychopathology. Introducing the training or manipulation to a clinical sample using rigorous designs and long-term follow-ups will help us disentangle whether DD is causally related to changes in health behaviors. Prospective longitudinal studies, such as the study by Audrain-McGovern et al. (2009), are another method to unravel the role of DD in psychopathology. As a final step, the same training or manipulation should be tested in a different clinical sample to deliver evidence for DD as transdiagnostic mechanism. This three-step approach would allow us to more efficiently test promising trainings and manipulations and provide evidence for the underlying theoretical framework of DD as transdiagnostic mechanism.

Secondly, we recommend extending the primary focus of this literature—to reduce DD rates and thereby change behavior—to an emphasis on the mechanisms that cause changes in DD and behavior. Although it is critical to test whether trainings or manipulations are able to effectively decrease DD and problematic behaviors, we also need to understand how and why they achieve this effect. Analyzing mechanisms of change, such as a focus on the valence of the future reward, can help us answer these how and why questions. Related to this, not only has heightened DD been correlated with health and disorder-related outcomes, but lower-than-average discount rates have also been associated with pathology (e.g., anorexia; Decker, Figner, & Steinglass, 2015). A focus on the broad range of discounting patterns and their possible effects on behavior is therefore recommended.

Thirdly, we want to make a call for an upgrade of the quality of studies and the sharing of measures and designs over studies. Differences between studies are high: Some studies did not include a proper control condition, some did not directly compare the experimental and control condition, and others included poorly powered designs or did not report effect sizes or statistics to calculate effect sizes. Furthermore, different methods were used to assess DD, and these methods vary systematically in their outcomes (Scheres, de Water, et al., 2013a; Weatherly, 2014). This is especially relevant as some of the “effective” trainings and manipulations in this systematic review have failed to replicate in more recent studies. For example, Zhang and Smith (2018) did not find an effect of power on DD in two preregistered, close-replication studies of Joshi and Fast’s study in 2013; Sweeney et al. (2018) failed to replicate the effects of a working memory training on DD in adolescents with cannabis use disorders; and Kable et al. (2017) showed that cognitive training (including working memory training) was unable to decrease DD in healthy controls. We recommend adopting a basic set of principles and methods based on transparency and replicability guidelines (Miguel et al., 2014; Simmons et al., 2011) which would upgrade the quality of studies in general and make it easier to perform replication studies and compare across studies.

Finally, we advise including more varied samples, in age as well as in clinical diagnosis, and including possible moderators of the effect of DD on behavior (e.g., motivation to change). For example, in the current systematic review, only two experiments included a sample younger than 18 years of age, with all other studies heavily relying on healthy control college student samples. From both a human impact and economic perspective, there is a strong rationale to focus more on youth (Coughlan et al., 2013; McGorry, 2013; McGorry, Purcell, Hickie, & Jorm, 2007; Patel, Flisher, Hetrick, & McGorry, 2007). As an example, we know that the major burden of smoking-related diseases falls on the adult population, but there are several reasons why smoking cessation among adolescents deserves special attention (Fanshawe et al., 2017). For example, smoking during adolescence has a direct negative effect on youth’s health and is a significant predictor of nicotine dependence in adulthood (Mermelstein, 2003), while without intervention, very few adolescent smokers quit smoking (Mermelstein, 2003). Therefore, delaying interventions until adulthood is undesirable, and a focus on youth is imperative.

### Conclusion

The current paper systematically reviewed behavioral trainings and manipulations that aimed to reduce DD in human participants. Overall, results showed that DD can be decreased and thus that DD is a changeable construct; manipulation effects on DD are easier demonstrated than effects of trainings. Most promising avenues to pursue in future research seem to be acceptance-based/mindfulness-based trainings, and even more so future-oriented manipulations. Furthermore, we call for more emphasis on the underlying theoretical framework of DD as transdiagnostic mechanism and more coherence in high-quality research methods across studies.
